# Deep-Manager: a versatile tool for optimal feature selection in live-cell imaging analysis

**DOI:** 10.1038/s42003-023-04585-9

**Published:** 2023-03-03

**Authors:** A. Mencattini, M. D’Orazio, P. Casti, M. C. Comes, D. Di Giuseppe, G. Antonelli, J. Filippi, F. Corsi, L. Ghibelli, I. Veith, C. Di Natale, M. C. Parrini, E. Martinelli

**Affiliations:** 1 Department of Electronic Engineering, University of Rome Tor Vergata, 00133 Rome, Italy; 2 Interdisciplinary Center for Advanced Studies on Lab-on-Chip and Organ-on-Chip Applications (IC-LOC), University of Rome Tor Vergata, 00133 Rome, Italy; 3 Department of Biology, University of Rome Tor Vergata, 00133 Rome, Italy; 4 Inserm U830, Stress and Cancer Lab, Institut Curie, Centre de Recherche, Paris Sciences et Lettres Research University, 75005 Paris, France

**Keywords:** Machine learning, Data processing

## Abstract

One of the major problems in bioimaging, often highly underestimated, is whether features extracted for a discrimination or regression task will remain valid for a broader set of similar experiments or in the presence of unpredictable perturbations during the image acquisition process. Such an issue is even more important when it is addressed in the context of deep learning features due to the lack of a priori known relationship between the black-box descriptors (deep features) and the phenotypic properties of the biological entities under study. In this regard, the widespread use of descriptors, such as those coming from pre-trained Convolutional Neural Networks (CNNs), is hindered by the fact that they are devoid of apparent physical meaning and strongly subjected to unspecific biases, i.e., features that do not depend on the cell phenotypes, but rather on acquisition artifacts, such as brightness or texture changes, focus shifts, autofluorescence or photobleaching. The proposed Deep-Manager software platform offers the possibility to efficiently select those features having lower sensitivity to unspecific disturbances and, at the same time, a high discriminating power. Deep-Manager can be used in the context of both handcrafted and deep features. The unprecedented performances of the method are proven using five different case studies, ranging from selecting handcrafted green fluorescence protein intensity features in chemotherapy-related breast cancer cell death investigation to addressing problems related to the context of Deep Transfer Learning. Deep-Manager, freely available at https://github.com/BEEuniroma2/Deep-Manager, is suitable for use in many fields of bioimaging and is conceived to be constantly upgraded with novel image acquisition perturbations and modalities.

## Abstract (web summary)

Deep-Manager is a software platform that enables efficient selection of features with lower sensitivity to unspecified disturbances across sets of similar experiments in bioimaging.

## Introduction

Reproducibility is a major concern in biomedical research, especially when it aims at building robust basis for future clinical therapies to improve human health. The biological data are often highly variable, mainly due to uncontrollable experimental parameters. This is particularly dramatic in the case of bioimage acquisitions for quantitative analysis. If images are not acquired on the same microscope, with the same setting, using the same light source and the same cell support, these images are not easily comparable unless standardization methods are implemented which, however, can alter the expected dynamics of the signals. This is a huge limitation in the application to biology of computational science methods, such as the powerful AI-based image analysis tools.

**Fig. 1:**
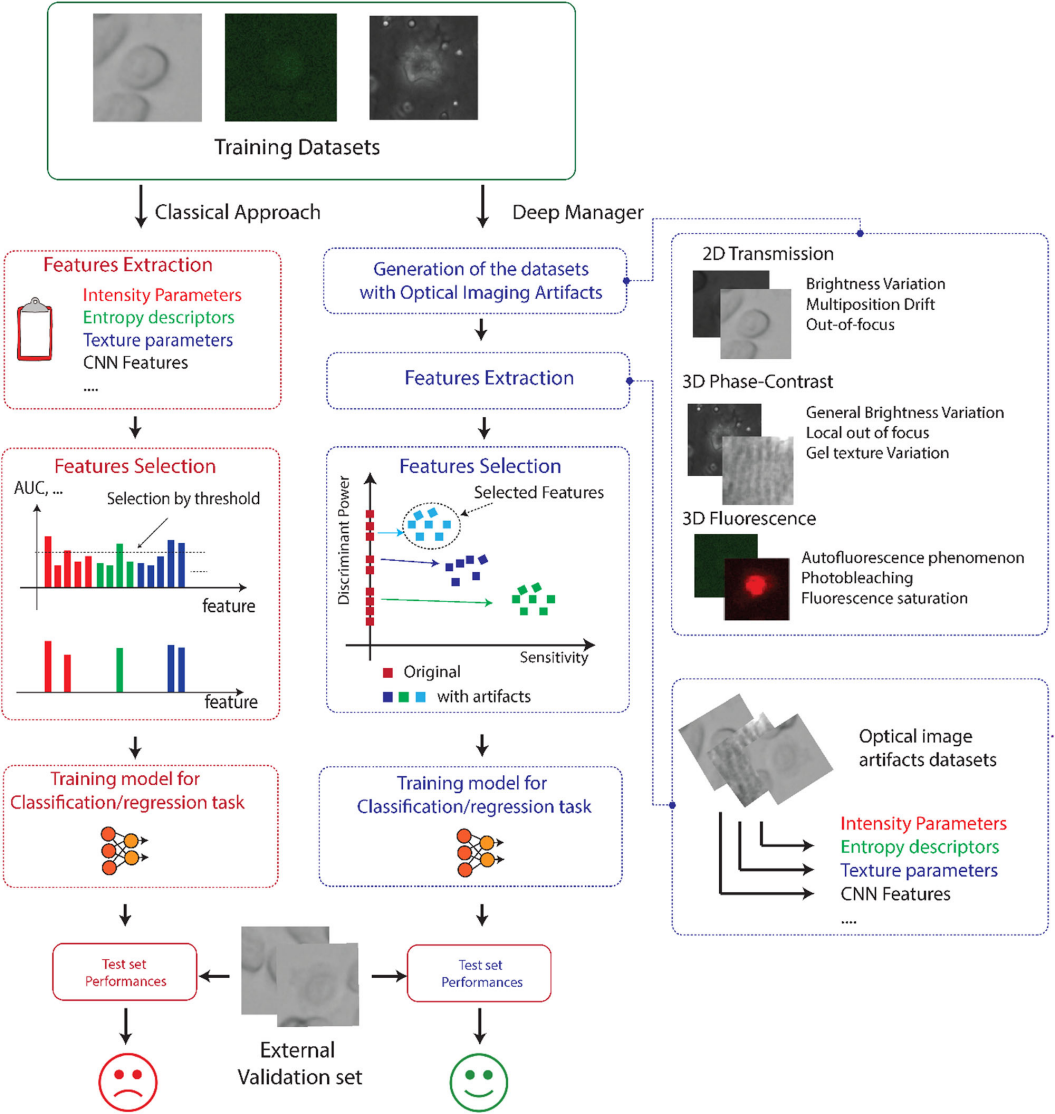
Architecture of the Deep-Manager platform. The red branch identifies the common practice in biomedical image analysis for feature selection. From top to bottom: extract intensity and texture descriptors, select features having the highest discrimination capability in terms of AUC, construct a classification model, test the performance over an external validation dataset. Blue branch identifies the Deep-Manager working flow. From top to bottom: modify the training dataset generating the optical imaging artifacts according to the imaging modality (right expansion), extract intensity, texture, or CNN features (deep features) from the modified dataset, calculate discrimination capability in terms of AUC (or DP) and evaluate the sensitivity of each feature by comparing the DP value before and after the perturbation, select those features with higher DP and lower sensitivity (cyan markers), construct a classification model over the selected features, and test on an external dataset of images. The robustness imposed in the feature selection step avoids a failure in the validation step and assures the construction of a more generally valid classification model.

In this regard, identifying a subset of image features that optimally relate to a specific disease or, more in general, to an aspect under investigation^1,2^ is still a frontier issue, often underestimated, especially in image-based classification tasks. The performance of classifiers run on a subset of handcrafted or black-box features is generally not scalable and usually sharply declines when used on datasets other than those used for classifier construction, lacking reproducibility and generalizability^3^. The main reason is that the experimental samples available for the feature selection step are usually scarce or not so general to cover possible admissible variations, even occurring within the same biological conditions. In practice, when the results obtained on a smaller set of experiments are extended to a more general and independent plethora of cases, the performance is expected to degrade dramatically, as shown in Fig. 1 (left, red branch). No matter if in the context of handcrafted or Deep Transfer Learning (DTL) features^4,5^ (i.e., descriptors coming from pre-trained Convolutional Neural Network (CNN)), it is essential to select the features that assure a very large validity over heterogeneous biological experiments, with appropriate representativeness and generalizability of the results. This aspect has been underestimated, especially in the context of DTL features, where two other major issues must be addressed: features dimensionality (thousands of features for a given image) and redundancy (many features are strongly correlated). The attention has been focused mainly on how to decrease the number of features to be extracted rather than on how to select the most general (i.e., valid) ones. The selection of the most representative descriptors, both handcrafted and DTL, in biomedical images is far from being an easy process and is highly prone to the risk that the features do not depend on the cell phenotypes but rather on brightness, texture artifacts, focus changes, autofluorescence, and other unpredictable disturbances. To solve this problem, we present here a platform, named Deep-Manager (DM) (the blue branch in Fig. 1), that allows to identify and practically select the best features for a given classification task after extraction by customized functions or after transfer by a given user-defined pre-trained DL network. The term *deep* refers explicitly to deep features, for which the problem of efficient feature selection is unsolved, and the risk of bias is huge^3^. However, as demonstrated in this work the platform may also run on handcrafted intensities and texture features commonly quantified in biomedical images. DM can therefore greatly help biologists in their everyday practice to verify the general validity of the rationally selected features. The DM platform identifies the extracted features that specifically represent the characteristics of the cell/tissue objects, discarding the non-specific macroscopic variations that unintentionally occur in the training dataset. This is crucial when the image acquisition process is very complex and at a practical limit of repeatability (e.g., *does the measured green emission intensity correlate with a specific event or simply with autofluorescence phenomena*? At the low-intensity level, the answer is not trivial). For example, in living cell biological experiments^6^, the acquisition process can be long (e.g., days), and the acquisition conditions are difficult to control for the entire period, both when using phase-contrast transmission light or fluorescence time-lapse (TM) microscopy^7,8^. The intra-experiment heterogeneity of video sequences, as well as the inter-experiment variation due to uncontrolled changes in the acquisition set-up^9^, also lead to high risks of wrong conclusions because of the low validity of the extracted features. These effects induce errors in the recognition model and misleading biological or clinical conclusions (e.g., not-true drug response). In this regard, the DM platform allows efficiently selecting, among all the features extracted from a DTL neural network or by customized handcrafted descriptors, those that have a lower sensitivity to disturbances and, at the same time, a high discriminating power (Fig. 1 blue branch). After the application of the different degradation tests to the training dataset (Fig. 1 right expansion), features are characterized in terms of their discriminant power (DP) and sensitivity to the degradations (SENS), measured as the relative difference in DP values before and after degradation injection (see Methods for the details). A multi-threshold approach is then used to separate features with high DP and low SENS (cyan dots in Fig. 1 blue branch) from the other groups of features (low DP or/and high sensitivity, green and blue dots in Fig. 1 blue branch). Selected features can then be used in a classification task proposed by the user, where it is asked to upload an independent test set of labeled images, the test dataset, to verify the validity of the selected features by evaluating their DP over a different set (Fig. 1).

The proposed approach is general and can be applied to any Deep Neural Network-based processing architecture by simply selecting a network among a proposed set of existing ones or, for users with programming skills, by modifying the Python software to integrate proprietary networks, new features, or also new perturbation tests. To demonstrate the usefulness of DM, we apply the software to five distinct case studies. Case study #1: *Fluorescence microscopy videos of chemotherapy-induced death of MDA-MB 231 breast cancer cells in the presence of a green fluorescent apoptosis reporter*^10^. Case study #2: *TL microscopy videos of PC3 human prostate cancer cells, moving in a 2D environment in the presence of the chemotherapeutic drug etoposide*^11^. Case study #3: *phase-contrast TL microscopy videos of immune cells moving in a 3D collagen gel inside microfluidic tumor-on-chip devices that mimic the tumor microenvironment*^12^. Case study #4: *fluorescence microscopy videos of cancer cells forced to undergo apoptosis by cytotoxic T cells in 3D tumor-on-chip*;^10^ Case study #5: *Phase contrast TL microscopy static images of BT-474 breast cancer cells from the recently presented public dataset LIVECell*^13^. In the following, we will refer to each case study simply using the case study number. In addition, for increased readability, we refer to the imaging modalities considered in the actual version of DM-tool as: *IM-ACQ-1* (2D TL time-lapse microscopy*), IM-ACQ-2* (3D phase-contrast TL time-lapse microscopy), *IM-ACQ-3* (3D fluorescence time-lapse microscopy). Each modality identifies specific tests related to the equipment and experimental conditions used as described below.

For all the five case studies considered, we obtained very good performance. In particular, when comparing the average sensitivity and the average discriminant power (DP) values of the features selected by DM with those obtained by the best comparative approach^14,15^ such as two-sample t-test with pooled variance estimate, Kullback-Leibler divergence, Chernoff bound, Mann–Whitney, area between the empirical receiver operating characteristic (ROC) curve, and stepwise linear regression fit, we obtained an improvement in DP and sensitivity values in the range 6–10% and 56–69% respectively with the additional strength of automatically providing a reduced set of selected features rather than a simple feature ranking. In addition, in case study 5, we also tested the discrimination capability of the selected features in an independent binary classification problem obtaining an average AUC of classification of 0.82 with an average AUC improvement with respect to a no-selection modality of 33%. The perturbation tests implemented are typical for each imaging modality (see Fig.1 right expansion) and can be further expanded according to additional perturbations of interest amp brightness drift, etc..). It is important to stress the fact that although real images present their own native artifacts due to the naturally imperfect acquisition protocol and sample heterogeneity, we need a controlled scenario in which we may quantify the effect of known source of image perturbations.

## Results and discussion

To demonstrate the effectiveness of the proposed Deep-Manager software platform, we selected five different use cases. The first use case concerns the exploitation of DM in extracting information from live-cell imaging, which is a common everyday practice among biologists. The clinical case refers to handcrafted intensity features related to GFP emission intensity in discriminating natural death from chemotherapy-induced death in MDA-MB 231 breast cancer cells^12^. The second use case concerns the extraction of robust deep features by DTL in TL microscopy videos of PC3 human prostate cancer cells in a 2D environment in the presence of the chemotherapeutic drug etoposide^11^. The third use case concerns deep features extraction in phase-contrast TL microscopy videos of immune cells moving in a 3D collagen gel inside microfluidic tumor-on-chip devices that mimic the tumor microenvironment^12^. The fourth use case considers the problem of extracting deep features in 3D fluorescence microscopy videos of cancer cells going into apoptosis due to the killing by cytotoxic T cells in 3D tumor-on-chip^12^. Finally, the fifth use addresses a classification task by using the deep features extracted using the DM platform. More specifically, we acquire phase contrast TL microscopy static images of BT-474 breast cancer cells from the recently published public dataset LIVECELL^13^, with the task to recognize the cancer growth factor after 4 h of culturing in a dish in a 5-day experiment. Details of each use case are provided in the next sections.

### Scores plot and classification results of the handcrafted features extracted (Case study #1)

In this case study, we compared the individual discrimination performance of handcrafted features GFP derived in terms of the p-value of the Student t-test with and without the selection using DM-tool. For the task of characterizing and discriminating between natural and chemotherapy-induced death, we selected four independent videos for each condition (8 in total), with a duration of 70 h, acquired at a time point of 1 h. We then automatically identified 431 crops, each containing a dying cell (i.e., a cell that is going to die within 70 h) through the use of the STAMP code^10^, downloadable at https://cloudstore.bee.uniroma2.it/index.php/s/LEpHYTsPnDj4Ajt (password: STAMP2021).

**Fig. 2:**
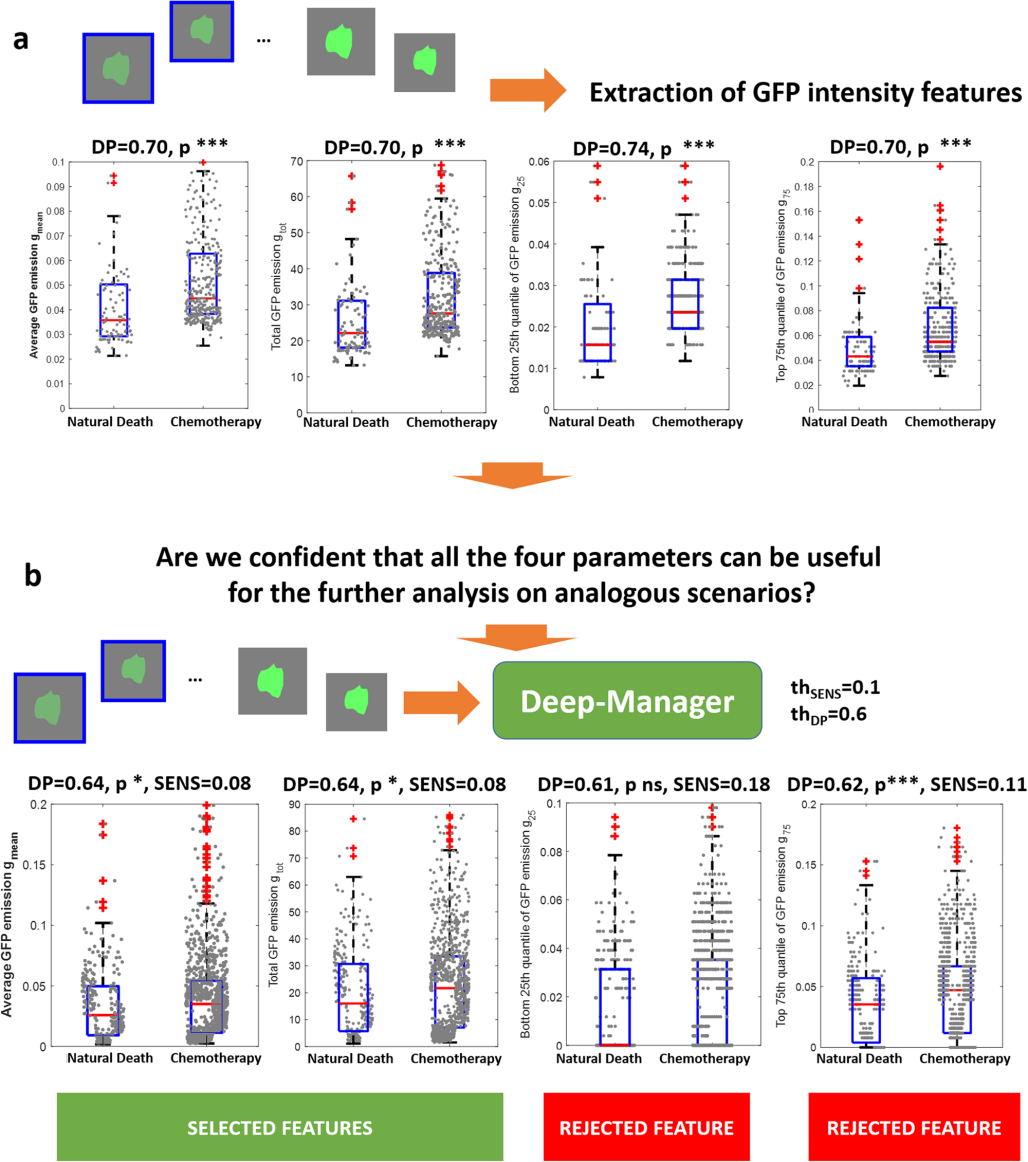
Case study #1. **a** A Standard feature extraction and discrimination capability evaluation using *p*-value for four handcrafted GFP intensity features {g_mean_, g_tot_, g_25_, and g_75_} computed over images acquired using fluorescence. *n* = 431 biologically independent samples have been considered. **b** Applying the DM platform to the four intensity features {g_mean_, g_tot_, g_25_, and g_75_} some descriptors reveal to be no more valid for the analysis, e,g,, g_75_, and g_25_ because too sensitive (SENS = 0.18 > th_SENS_ (0.1) and SENS = 0.11 > th_SENS_ (0.1) respectively) to perturbations (autofluorescence, photobleaching, saturation). Nevertheless, descriptor g75, still remains significant in terms of t-test analysis (*p*-value < ***) but presents a sensitivity value SENS larger than the threshold due to an unacceptable worsening in the DP performance after perturbation injection. *n* = 1293 biologically independent samples have been considered.

In particular, 96 crops are from natural death, and 335 crops are from cytotoxic T-cell death. Further details of the image processing step can be found in^10^. Each crop has been characterized in terms of the following handcrafted features: average green emission in the crop, *g*_mean_, the total green emission in the crop *g*_tot_, the top 75th percentile of the green emission in the crop *g*_75_, the bottom 25th percentile green emission in the crop *g*_25_. Usually, the percentiles 25th and 75th values are preferred to *max* and *min* that are unpredictably subjected to occasional disturbances. The discrimination capability of the features computed on the available crops is evaluated in terms of the individual DP (max(1-AUC,AUC)) and in terms of the *p*-value of a paired two-sample Student t-test (see Eqs. (6), (7) in the Supplementary Information). Results are shown in Fig. 2, panel A. Preliminary evidence demonstrated the discriminability of the two death phenomena by using all four descriptors. A DP value was equal to or higher than 0.70, and the *p-*value was below 0.005. To be more confident of the results, we then applied DM platform to the crops in the training set. By selecting the right scenario (3D fluorescence microscopy), we applied perturbation tests related to autofluorescence, photobleaching, and saturation and calculated the four descriptors {*g*_mean_, *g*_tot_, *g*_75_, and *g*_25_} over the modified images. The DP values of the descriptors are then compared with those obtained before image alterations, and the sensitivity SENS is calculated for each feature. Figure 2 panel b shows the new boxplots of the four descriptors computed over the modified set of images, with the corresponding DP, SENS, and *p-*value indicated. As it is immediately evident, while descriptors {*g*_mean_, *g*_tot_} still remain acceptably robust, DP decreases (but over the limit threshold of 0.6) but with a percentage change (SENS) that is less than the limit value of 0.1. On the contrary, descriptors {*g*_75_, and *g*_25_} that are often used in the discrimination of death events, present a strong decrease in the DP values: *g*_25_ DP values goes from 0.75 to 0.61 with a percentage sensitivity SENS equal to 0.18 far beyond the limit value of 0.1 and *g*_75_ DP values goes from 0.70 to 0.62 with a percentage sensitivity SENS equal to 0.11 still beyond the limit value of 0.1. One of the most astonishing fact is also the permanence of statistical significance in terms of *p-*value for descriptor *g*_75_. In other words, the DM platform allows the use of realizing which descriptors have chances to be valid for a larger plethora of experiments and with an increased complexity of biological phenomena, relaxing the absolute trust in the training set but rather as a subset of a wider and representative set.

### Scores plot result for the extracted features for Case studies 2–4

**Fig. 3:**
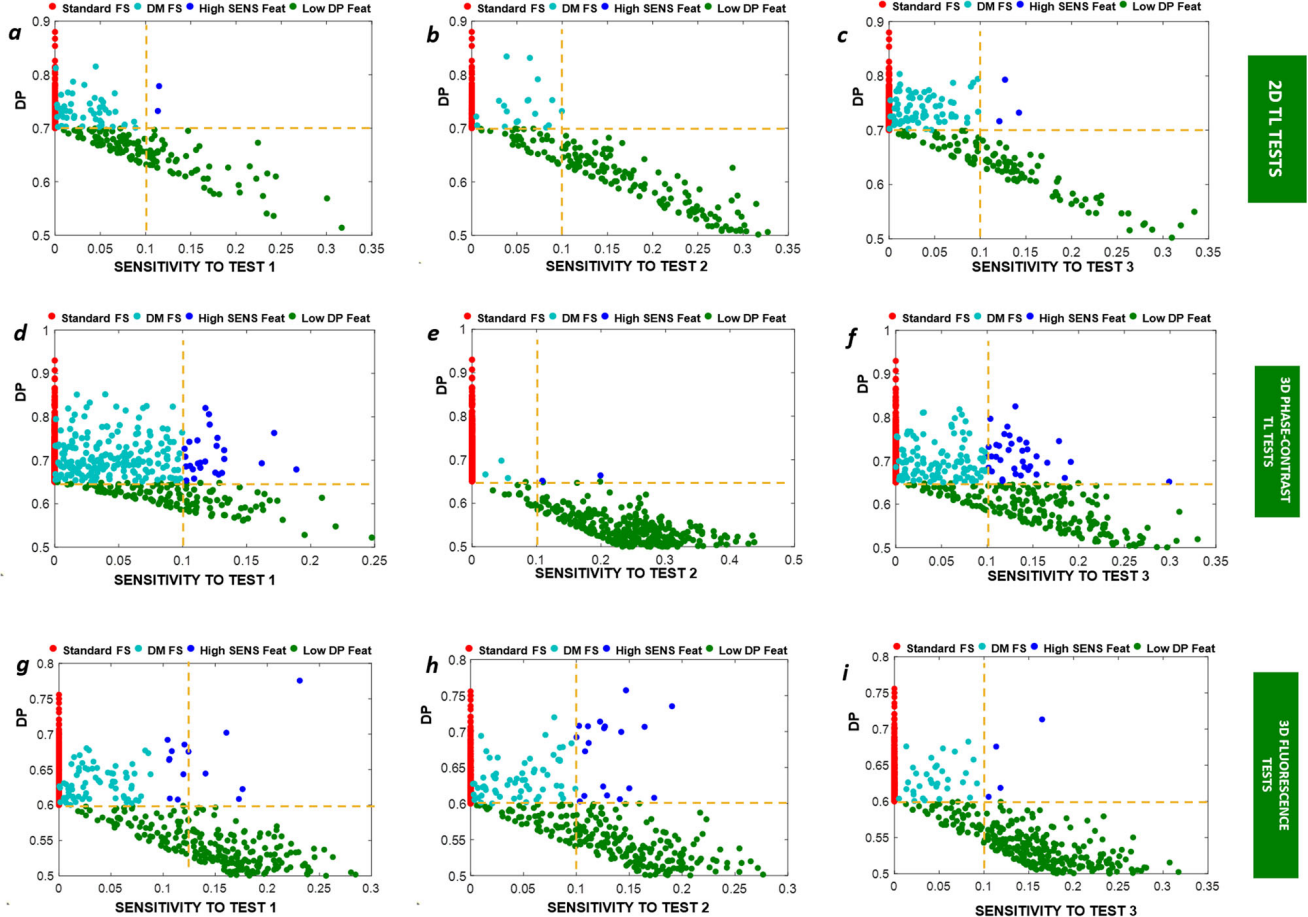
SENS vs DP plot. Score plots (SENS, DP) of the feature extracted using transfer learning (‘Resnet101’ is used in the example) for **a**–**c** Case study #2, **d**–**f** Case study #3, **g**–**I** Case study #4 and for the three tests for each modality, **a**, **d**, **g** test 1, **b**, **e**, **h** test2, **c**, **f**, **i** test3. Cyan markers locate the (SENS, DP) values for each selected descriptor (high DP and low SENS). Blue markers locate the (SENS, DP) values for each descriptor rejected due to a too high SENS. Green markers locate the (SENS, DP) values for rejected descriptors due to a too low DP.

In this task, we demonstrated the working principle of the DM-tool over case studies 2–4, by splitting, for each imaging modality and each test applied, the deep features extracted according to the sensitivity and DP values. In Fig. 3a–c, we show the scores plots (SENS, DP) for each feature extracted: red markers indicate the DP of the features without the sensitivity test (assumed SENS = 0), cyan markers indicate the score plot of the selected features, blue and green ones are those rejected (high SENS and low DP respectively). The orange lines indicate the threshold values for DP (horizontal line) and for the SENS (vertical line). Plots (a) to (c) indicate the three case studies respectively, for each test (from left to right). The number of selected features is also indicated.

### Comparison with standard feature selection approaches

In this task, we compared the DM-tool feature selection performance with other existing selection methods. To avoid the compensation effect with a classification model trained over the selected features and to appreciate the robustness of the features selected using the proposed tool, we only compared the sensitivity and DP values of the features selected using the approaches. With the aim of comparing different standard feature selection approaches with DM selection, we proceed as follows. First, we extracted the features from the original training dataset of images by performing transfer learning from a given CNN and related pooling layer. Then, we ranked the features according to a comparative method among those listed below and kept the first *N*_sel_ where *N*_sel_ is the number of features selected by DM. We then extract the DP and the SENS values for the selected features when computed over the artifacted dataset and use them for comparison. In this way, we verify the potential of selecting features using the sensitivity criterion in addition to the DP. For clarity, also METHOD 5 uses the DP criterion for ranking but without the evaluation of feature sensitivity. As it will be shown below the DP criterion obtains very low performance if used alone. In particular, we compared with the following methods: *rankfeature* based on features ranking by class separability criteria^15^ and stepwise regression model^14^.

More in details, we consider:

- METHOD 1: rank feature by using criteria such as: *t-test*, namely the absolute value two-sample t-test with pooled variance estimate^15^.
- METHOD 2: rank feature by using criteria such as: *entropy*, namely relative entropy (Kullback–Leibler divergence)^15^.
- METHOD 3: rank feature by using criteria such as: *Bhattacharyya*, namely the minimum attainable classification error or Chernoff bound^15^.
- METHOD 4: rank feature by using criteria such as: *Wilcoxon*, namely absolute value of the standardized u-statistic of a two-sample unpaired Wilcoxon test, also known as Mann-Whitney^15^.
- METHOD 5: rank feature by using criteria such as: *ROC*, area between the empirical ROC curve and the random classifier slope^15^.
- METHOD 6: stepwise linear regression fit for feature selection^14^.

**Fig. 4:**
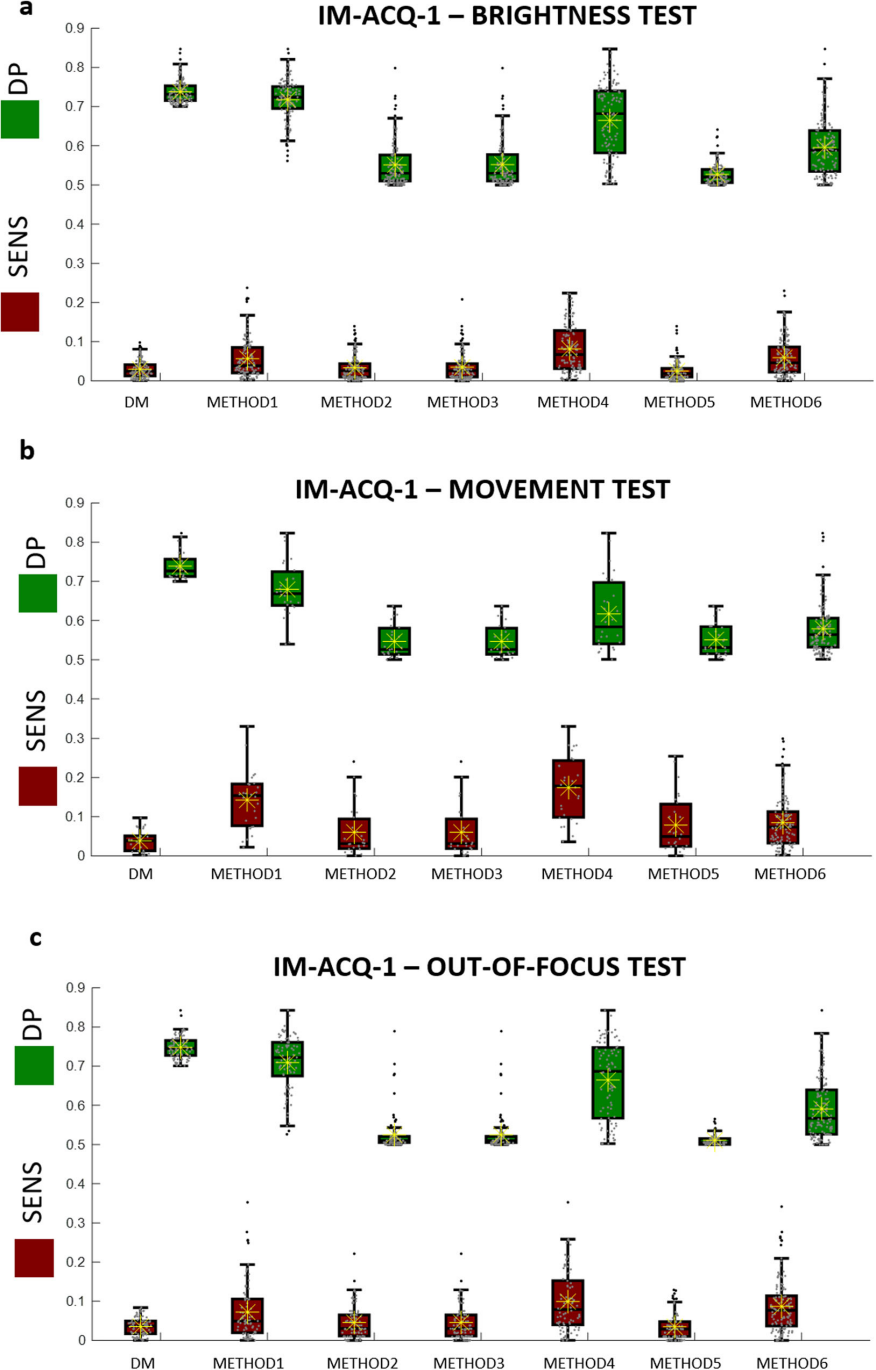
Boxplots of the SENS values (orange boxes) and DP values (green boxes) for 6 comparative approaches, METHODS 1–6, and the proposed Deep-Manager approach (first result). Yellow asterisks identify the average values and the horizontal black line identifies the median value. Results are for Case study #2, IM-ACQ-1, tests 1(**a**)−3(**c**). *n* = 200 biologically independent samples have been considered.

**Fig. 5:**
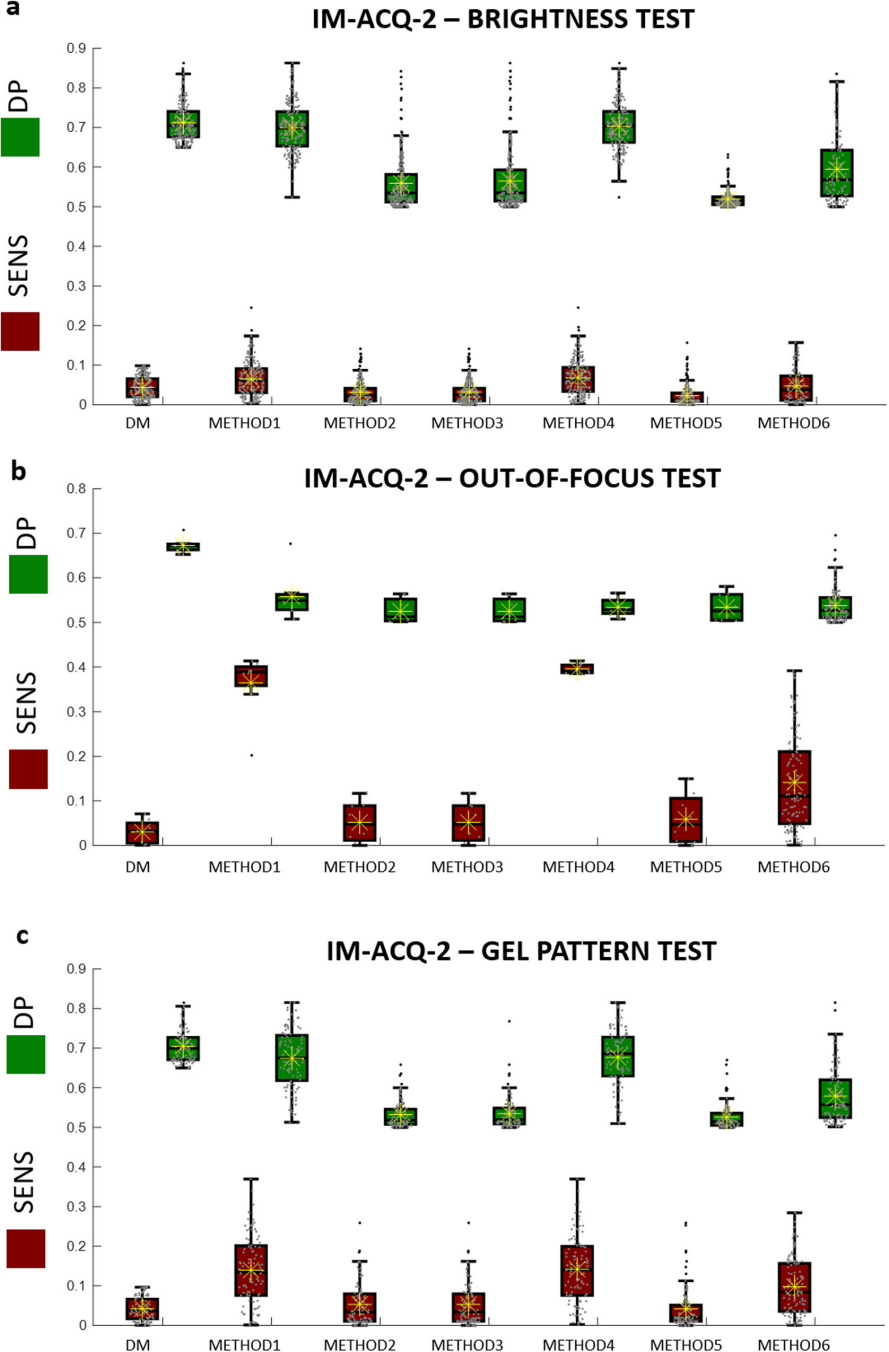
Boxplots of the SENS values (orange boxes) and DP values (green boxes) for 6 comparative approaches, METHODS 1–6, and the proposed Deep-Manager approach (first result). Yellow asterisks identify the average values and the horizontal black line identifies the median value. Results are for Case study #3, IM-ACQ-2, tests 1(**a**)−3(**c**). *n* = 200 biologically independent samples have been considered.

**Fig. 6:**
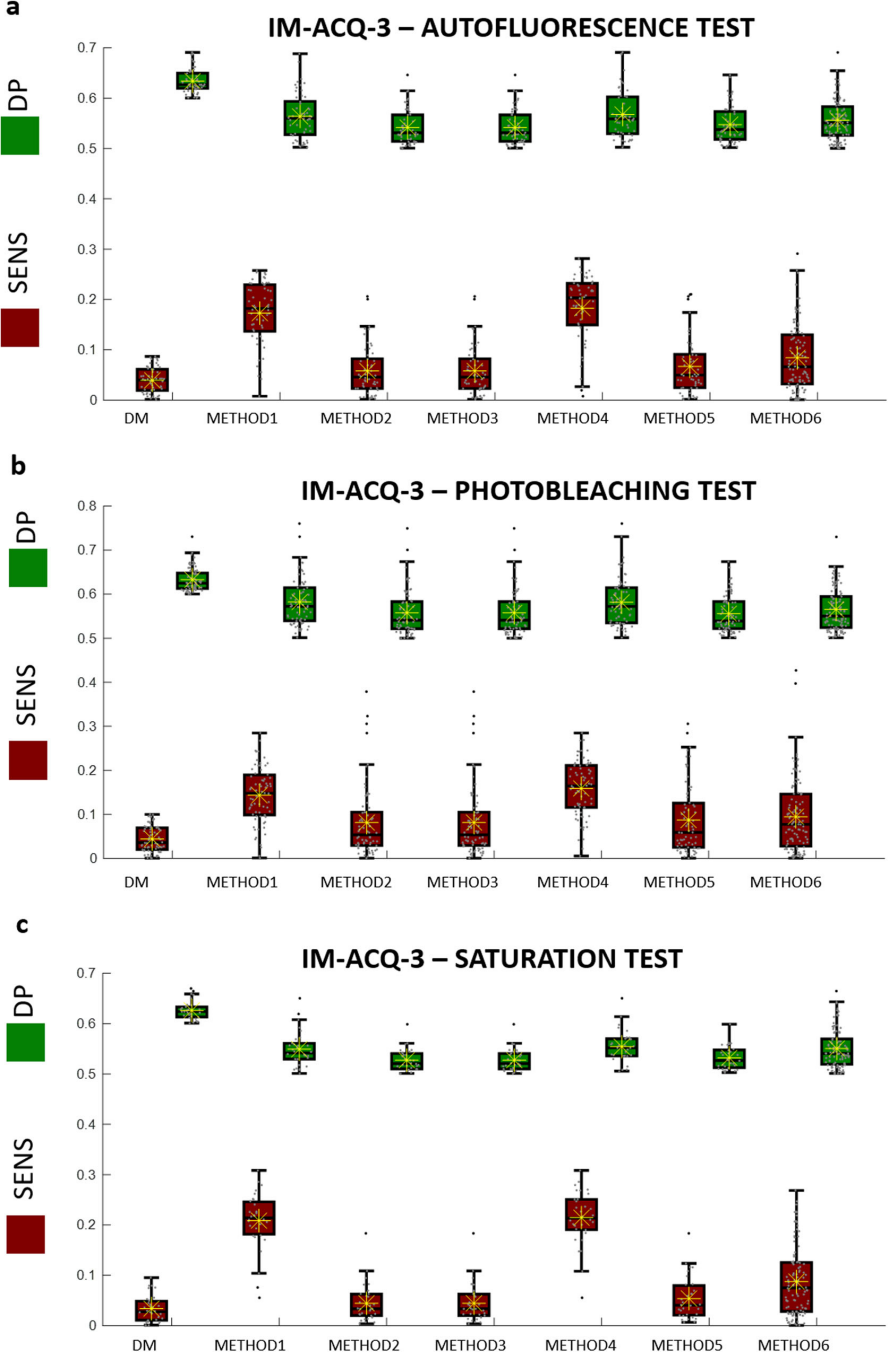
Boxplots of the SENS values (orange boxes) and DP values (green boxes) for 6 comparative approaches, METHODS 1–6, and the proposed Deep-Manager approach (first result). Yellow asterisks identify the average values and the horizontal black line identifies the median value. Results are for Case study #4, IM-ACQ-3, tests 1(**a**)−3(**c**). *n* = 200 biologically independent samples have been considered.

For each subset of selected features, we evaluate the score plot (SENS, DP) by calculating the same features over the artifacted set of images. Figures 4–6 illustrate boxplot (orange boxes for SENS, green boxes for DP values) for the selected features according to the method used for the selection. Paired boxplots indicated the (SENS,DP) distribution values for each method. As it can be observed, METHOD 1 (t-test) and METHOD 4 (Wilcoxon test) often demonstrate to reach good performance but exhibit a spreader distribution of values for DP (green boxes) and for SENS (orange boxes). On the other hand, METHOD 2 (entropy), METHOD 3 (*Bhattacharyya)*, METHOD 5 (ROC), and METHOD 6 (stepwise linear fit) exhibit unacceptably low DP values even if at very low SENS values. The results are confirmed for all the tests (TESTS 1–3) and for all the case studies (case studies 2, 3, and 4).

### Comparison of different Deep Learning networks

In order to furthermore demonstrate the effectiveness of Deep-Manager feature selection approach, we compare different Deep Learning network architectures among those most used. Again, we did not implement a classification step over the selected features to avoid masking the robustness of the features selected. Comparison will be performed in terms of sensitivity and DP values. In particular, we considered ResNET10116, VGG1917, NasNETLarge18, and DenseNET20119. Layers used for each architecture are selected as a trade-off between memory storage and time-consuming (deeper layers provide a coarser representation of the image, therefore, extracting less descriptors) and discrimination performance. Table 1 lists the layers used and the number of descriptors considered for the analysis. Tables 2–4 show the numerical results of DP and SENS of the features selected by each comparative method (columns 2–7) and the DM tool (first column), by using the networks and the layers listed in Table 1, for case studies #2–#4. METHODS 1–6 are applied as follows. First, features are extracted by applying a given network and layer over the original dataset of training images. Then, features are ranked according to the criterion included in methods (1–5) or directly selected using method 6. After ranking, features are selected by taking the first Nsel ranked features, where Nsel is the number of features selected using DM tool. By extracting also the same features over the modified dataset of images (obtained after applying the proper tests according to the imaging modality), we then computed the SENS and the new DP that are listed in Table 2. In this way, we may directly evaluate the importance of using the sensitivity criterion in conjunction with the DP value, especially in comparing DM approach with the METHOD 5 that actually uses a DP-like ranking criterion, but evaluate on the original dataset and without the support of the sensitivity evaluation procedure. Average values of SENS and DP are listed in the table, while values in brackets represent the standard deviation computed over the set of features selected. Best results for each test are bolded. As it can be seen, in all the 36 tests, Deep-Manager achieves higher or equivalent DP values for the selected features. Noteworthy is the fact that METHODS 1–5 do not autonomously provide a subset of selected features but rather a feature ranking result. Therefore, METHODS 1–5 would require a further optimization step for feature selection. On the contrary, METHOD 6 (stepwise linear regression) that actually returns a subset of selected features never provides acceptable results in terms of the DP of the extracted features when performances are calculated over the features extracted in the modified set of images.

**Table 1:** Lists of deep learning architectures selected for the validation and related characteristics: layers used and total number of features extracted.

	ResNET101 He et al., 2016	VGG19 A Bhandary et al., 2020	NasNETLarge Zoph et al., 2018	DenseNET201 Huang et al, 2017
Layer	‘average-pool5’	‘max-pool5’	‘global_average_pool5’	‘average_pool5’
N. of features	2048	25088	4032	1920
Input Layer size	224 × 224 × 3	224 × 224 × 3	331 × 331 × 3	224 × 224 × 3

**Table 2:** Numerical results in terms of average sensitivity, SENS, and DP values (standard deviation) for Deep-Manager (DM) and METHODS 1–6 for Case Study 2 using network architectures ResNET101, VGG19, NasNETLarge, and DenseNET.

CASE STUDY 2														
RESNET101														
	DM		METHOD 1		METHOD2		METHOD3		METHOD4		METHOD5		METHOD6	
	SENS	DP	SENS	DP	SENS	DP	SENS	DP	SENS	DP	SENS	DP	SENS	DP
TEST1	0.03 (0.02)	0.74 (0.03)	0.06 (0.05)	0.72 (0.05)	0.03 (0.03)	0.55 (0.06)	0.03 (0.03)	0.56 (0.06)	0.08 (0.06)	0.67 (0.09)	0.02 (0.03)	0.53 (0.03)	0.06 (0.05)	0.60 (0.07)
TEST2	0.04 (0.03)	0.74 (0.03)	0.14 (0.07)	0.68 (0.07)	0.06 (0.06)	0.55 (0.04)	0.06 (0.06)	0.55 (0.04)	0.17 (0.08)	0.62 (0.09)	0.08 (0.07)	0.55 (0.04)	0.08 (0.07)	0.58 (0.06)
TEST3	0.04 (0.02)	0.75 (0.03)	0.07 (0.07)	0.71 (0.07)	0.05 (0.04)	0.52 (0.05)	0.05 (0.04)	0.52 (0.05)	0.10 (0.08)	0.66 (0.10)	0.03 (0.03)	0.51 (0.01)	0.09 (0.07)	0.59 (0.08)
VGG19														
TEST1	0.03 (0.02)	0.72 (0.02)	0.05 (0.04)	0.71 (0.03)	0.01 (0.01)	0.51 (0.01)	0.01 (0.01)	0.51 (0.01)	0.06 (0.05)	0.70 (0.04)	0.01 (0.01)	0.51 (0.01)	0.02 (0.03)	0.55 (0.06)
TEST2	0.03 (0.02)	0.73 (0.02)	0.11 (0.10)	0.67 (0.08)	0.03 (0.04)	0.52 (0.02)	0.12 (0.10)	0.66 (0.08)	0.20 (0.08)	0.61 (0.08)	0.02 (0.03)	0.51 (0.02)	0.05 (0.06)	0.55 (0.05)
TEST3	0.05 (0.03)	0.75 (0.03)	0.10 (0.02)	0.68 (0.08)	0.02 (0.02)	0.50 (0.00)	0.02 (0.02)	0.55 (0.00)	0.10 (0.08)	0.69 (0.08)	0.01 (0.01)	0.50 (0.00)	0.07 (0.07)	0.55 (0.06)
NASNETLARGE														
TEST1	0.05 (0.03)	0.72 (0.01)	0.11 (0.02)	0.70 (0.02)	0.02 (0.01)	0.52 (0.01)	0.02 (0.01)	0.52 (0.01)	0.12 (0.02)	0.70 (0.02)	0.06 (0.04)	0.59 (0.05)	0.04 (0.03)	0.60 (0.08)
TEST2	0.03 (0.02)	0.73 (0.02)	0.21 (0.09)	0.62 (0.07)	0.02 (0.01)	0.52 (0.01)	0.02 (0.01)	0.52 (0.01)	0.18 (0.09)	0.64 (0.06)	0.02 (0.02)	0.51 (0.01)	0.09 (0.07)	0.55 (0.04)
TEST3	0.01 (0.01)	0.73 (0.02)	0.01 (0.01)	0.72 (0.03)	0.01 (0.01)	0.51 (0.01)	0.02 (0.01)	0.51 (0.01)	0.10 (0.01)	0.72 (0.03)	0.01 (0.01)	0.50 (0.01)	0.02 (0.02)	0.59 (0.07)
DENSENET201														
TEST1	0.06 (0.03)	0.73 (0.02)	0.20 (0.06)	0.67 (0.06)	0.09 (0.03)	0.56 (0.02)	0.09 (0.03)	0.56 (0.02)	0.17 (0.06)	0.69 (0.05)	0.09 (0.03)	0.56 (0.02)	0.13 (0.07)	0.59 (0.05)
TEST2	0.03 (0.02)	0.73 (0.02)	0.10 (0.10)	0.69 (0.07)	0.06 (0.06)	0.62 (0.09)	0.07 (0.07)	0.62 (0.09)	0.12 (0.10)	0.68 (0.07)	0.05 (0.05)	0.55 (0.04)	0.11 (0.08)	0.61 (0.06)
TEST3	0.03 (0.02)	0.75 (0.02)	0.04 (0.04)	0.74 (0.04)	0.05 (0.05)	0.67 (0.10)	0.05 (0.05)	0.74 (0.04)	0.04 (0.04)	0.60 (0.10)	0.06 (0.05)	0.60 (0.10)	0.06 (0.05)	0.67 (0.09)

**Table 3:** Numerical results in terms of average DP and sensitivity (SENS) values (standard deviation) for Deep-Manager (DM) and METHODS 1–6 for Case Study 3 using network architectures ResNET101, VGG19, and NasNETLarge.

CASE STUDY 3														
RESNET101														
	DM		METHOD 1		METHOD2		METHOD3		METHOD4		METHOD5		METHOD6	
	SENS	DP	SENS	DP	SENS	DP	SENS	DP	SENS	DP	SENS	DP	SENS	DP
TEST1	0.04 (0.03)	0.71 (0.05)	0.07 (0.05)	0.70 (0.06)	0.04 (0.03)	0.56 (0.07)	0.04 (0.03)	0.57 (0.07)	0.07 (0.05)	0.70 (0.06)	0.03 (0.03)	0.52 (0.02)	0.05 (0.04)	0.60 (0.08)
TEST2	0.04 (0.02)	0.67 (0.02)	0.43 (0.01)	0.51 (0.01)	0.05 (0.02)	0.53 (0.01)	0.05 (0.02)	0.53 (0.01)	0.43 (0.01)	0.52 (0.01)	0.04 (0.02)	0.53 (0.01)	0.14 (0.11)	0.54 (0.03)
TEST3	0.05 (0.03)	0.70 (0.04)	0.13 (0.07)	0.68 (0.07)	0.04 (0.04)	0.54 (0.05)	0.04 (0.04)	0.54 (0.05)	0.13 (0.07)	0.68 (0.06)	0.03 (0.04)	0.52 (0.02)	0.09 (0.07)	0.58 (0.07)
VGG19														
TEST1	0.03 (0.02)	0.70 (0.03)	0.05 (0.04)	0.67 (0.05)	0.01 (0.01)	0.51 (0.02)	0.01 (0.01)	0.51 (0.02)	0.05 (0.04)	0.68 (0.04)	0.01 (0.01)	0.51 (0.01)	0.04 (0.04)	0.57 (0.08)
TEST2	0.04 (0.03)	0.70 (0.04)	0.29 (0.08)	0.55 (0.07)	0.03 (0.04)	0.51 (0.02)	0.03 (0.04)	0.51 (0.02)	0.29 (0.08)	0.56 (0.06)	0.02 (0.03)	0.51 (0.01)	0.11 (0.10)	0.54 (0.04)
TEST3	0.04 (0.03)	0.69 (0.03)	0.07 (0.05)	0.67 (0.06)	0.02 (0.02)	0.52 (0.02)	0.02 (0.02)	0.52 (0.02)	0.06 (0.05)	0.68 (0.05)	0.01 (0.01)	0.51 (0.02)	0.04 (0.04)	0.57 (0.07)
NASNETLARGE														
TEST1	0.05 (0.03)	0.70 (0.04)	0.27 (0.07)	0.61 (0.07)	0.09 (0.09)	0.53 (0.04)	0.09 (0.09)	0.53 (0.04)	0.30 (0.07)	0.58 (0.06)	0.02 (0.03)	0.50 (0.01)	0.14 (0.10)	0.57 (0.05)
TEST2	0.04 (0.03)	0.75 (0.05)	0.06 (0.05)	0.74 (0.07)	0.06 (0.06)	0.70 (0.10)	0.06 (0.06)	0.71 (0.10)	0.06 (0.06)	0.73 (0.08)	0.05 (0.05)	0.70 (0.11)	0.08 (0.07)	0.65 (0.10)
TEST3	0.03 (0.01)	0.74 (0.05)	0.03 (0.03)	0.73 (0.06)	0.04 (0.03)	0.69 (0.10)	0.04 (0.03)	0.69 (0.10)	0.03 (0.03)	0.74 (0.06)	0.03 (0.03)	0.70 (0.10)	0.05 (0.04)	0.64 (0.10)
DENSENET201														
TEST1	0.04 (0.03)	0.71 (0.04)	0.13 (0.08)	0.67 (0.07)	0.11 (0.07)	0.64 (0.08)	0.11 (0.07)	0.64 (0.08)	0.14 (0.08)	0.67 (0.07)	0.13 (0.08)	0.67 (0.08)	0.09 (0.07)	0.60 (0.07)
TEST2	0.05 (0.03)	0.71 (0.04)	0.18 (0.11)	0.65 (0.09)	0.16 (0.10)	0.64 (0.08)	0.17 (0.10)	0.64 (0.08)	0.19 (0.10)	0.64 (0.09)	0.19 (0.11)	0.64 (0.09)	0.17 (0.12)	0.64 (0.09)
TEST3	0.03 (0.02)	0.72 (0.05)	0.05 (0.05)	0.71 (0.06)	0.05 (0.04)	0.67 (0.09)	0.05 (0.04)	0.68 (0.09)	0.05 (0.04)	0.71 (0.06)	0.05 (0.05)	0.71 (0.06)	0.06 (0.04)	0.62 (0.08)

**Table 4:** Numerical results in terms of average DP and sensitivity (SENS) values (standard deviation) for Deep-Manager (DM) and METHODS 1–6 for Case Study 4 using network architectures ResNET101, VGG19, NasNETLarge, and DenseNET201.

CASE STUDY 4														
RESNET101														
	DM		METHOD 1		METHOD2		METHOD3		METHOD4		METHOD5		METHOD6	
	SENS	DP	SENS	DP	SENS	DP	SENS	DP	SENS	DP	SENS	DP	SENS	DP
TEST1	0.04 (0.03)	0.71 (0.04)	0.07 (0.05)	0.70 (0.06)	0.04 (0.03)	0.56 (0.07)	0.04 (0.03)	0.56 (0.07)	0.07 (0.05)	0.70 (0.06)	0.03 (0.03)	0.52 (0.02)	0.05 (0.04)	0.59 (0.08)
TEST2	0.05 (0.03)	0.64 (0.03)	0.15 (0.06)	0.58 (0.05)	0.09 (0.08)	0.56 (0.05)	0.09 (0.08)	0.56 (0.05)	0.16 (0.07)	0.58 (0.05)	0.09 (0.08)	0.56 (0.05)	0.10 (0.08)	0.57 (0.05)
TEST3	0.05 (0.02)	0.63 (0.02)	0.20 (0.08)	0.56 (0.05)	0.05 (0.04)	0.53 (0.02)	0.05 (0.04)	0.53 (0.02)	0.22 (0.07)	0.53 (0.03)	0.06 (0.05)	0.53 (0.03)	0.09 (0.07)	0.56 (0.04)
VGG19														
TEST1	0.03 (0.02)	0.69 (0.03)	0.05 (0.04)	0.67 (0.05)	0.01 (0.01)	0.51 (0.02)	0.01 (0.01)	0.51 (0.02)	0.05 (0.04)	0.68 (0.04)	0.01 (0.01)	0.51 (0.01)	0.04 (0.04)	0.57 (0.08)
TEST2	0.04 (0.03)	0.64 (0.03)	0.18 (0.08)	0.57 (0.06)	0.03 (0.04)	0.52 (0.03)	0.03 (0.04)	0.52 (0.03)	0.20 (0.08)	0.57 (0.05)	0.02 (0.04)	0.51 (0.02)	0.10 (0.08)	0.53 (0.04)
TEST3	0.04 (0.03)	0.63 (0.03)	0.22 (0.07)	0.56 (0.05)	0.03 (0.03)	0.52 (0.02)	0.03 (0.03)	0.52 (0.01)	0.23 (0.07)	0.56 (0.05)	0.03 (0.04)	0.51 (0.02)	0.10 (0.08)	0.53 (0.04)
NASNETLARGE														
TEST1	0.05 (0.03)	0.70 (0.03)	0.27 (0.07)	0.61 (0.07)	0.09 (0.09)	0.53 (0.04)	0.09 (0.09)	0.53 (0.04)	0.30 (0.07)	0.59 (0.07)	0.02 (0.03)	0.50 (0.01)	0.14 (0.09)	0.57 (0.05)
TEST2	0.04 (0.08)	0.56 (0.05)	0.10 (0.08)	0.55 (0.04)	0.10 (0.08)	0.55 (0.04)	0.10 (0.08)	0.55 (0.04)	0.22 (0.08)	0.57 (0.05)	0.08 (0.09)	0.54 (0.04)	0.10 (0.09)	0.56 (0.05)
TEST3	0.04 (0.03)	0.64 (0.03)	0.23 (0.07)	0.56 (0.05)	0.10 (0.08)	0.55 (0.04)	0.11 (0.08)	0.55 (0.04)	0.24 (0.07)	0.54 (0.05)	0.09 (0.08)	0.55 (0.04)	0.11 (0.08)	0.55 (0.04)
DENSENET201														
TEST1	0.04 (0.03)	0.71 (0.04)	0.13 (0.08)	0.67 (0.07)	0.11 (0.07)	0.64 (0.08)	0.11 (0.07)	0.64 (0.08)	0.14 (0.08)	0.67 (0.07)	0.13 (0.08)	0.67 (0.08)	0.09 (0.07)	0.60 (0.07)
TEST2	0.04 (0.03)	0.64 (0.03)	0.20 (0.08)	0.57 (0.06)	0.10 (0.08)	0.57 (0.04)	0.10 (0.08)	0.57 (0.04)	0.21 (0.08)	0.57 (0.05)	0.21 (0.08)	0.57 (0.06)	0.11 (0.08)	0.56 (0.05)
TEST3	0.04 (0.03)	0.62 (0.02)	0.26 (0.05)	0.55 (0.04)	0.09 (0.06)	0.54 (0.03)	0.09 (0.06)	0.54 (0.03)	0.25 (0.07)	0.56 (0.04)	0.25 (0.07)	0.56 (0.04)	0.08 (0.07)	0.55 (0.04)

### Performances comparison of different layers for the same network

**Fig. 7:**
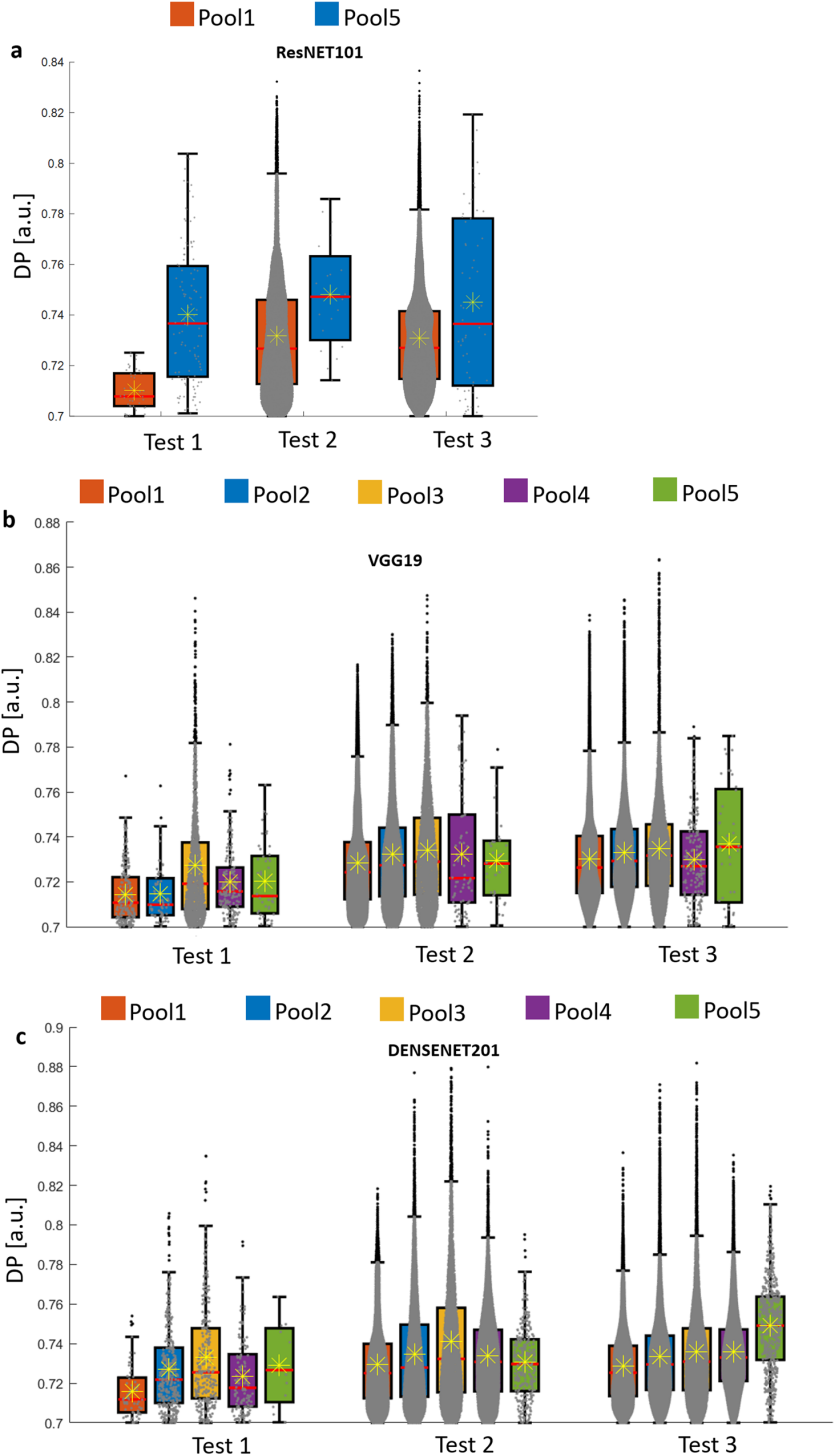
Performance in terms of DP values by changing the deep layer for transfer learning. **a** ResNET101, max-pool1, avg-pool5. **b** VGG19, map-pool1, max-pool2, max-pool3, max-pool4, max-pool5. **c** DenseNET201, max-pool1, avg-pool2, avg-pool3, avg-pool4, and avg-pool5. *n* = 46770 independent data have been used for panel (**a**), *n* = 127667 independent data have been used for panel (**b**), *n* = 26675 independent data have been used for panel (**c**).

Convolutional Neural networks are composed of repeated nested convolution operations alternated with nonlinear operations and pooling layers. It is quite unpredictable which layers to use for optimal transfer learning implementation. Usually, pooling layers are preferred due to the fact that they return a more compact set of information with respect to any previous convolutional layers. With the aim to presenting a further potential of Deep-Manager platform, we compare the DP values for features selected from different layers of diverse CNNs. Results are provided for the case study 2 (2D culture) using networks ResNET101 (max-pool1 and avg-pool5), VGG19 (max-pool1, max-pool2, max-pool3, max-pool4, max-pool5) and DenseNET201 (max-pool1, avg-pool2, avg-pool3, avg-pool4, avg-pool5). NasNetLarge is not represented here since it presents a unique pooling layer (i.e., global_average pool5). Figure 7 compares the DP distribution of features selected from each layer. Previous experiments highlight the importance of selecting the correct pooling layer. As it can be seen the DP values of the selected features depend on the layer and on the test applied. On average, it may be noticed that a middle layer (pool3 for DenseNET201 and for VGG19) reaches the highest DP values for test 1 and test 2 (case study 2). On the contrary, pool5 reaches the best performance in the average DP values for test 3 (case study 2). However, this fact should be correlated with a highly lower number of features to manage as long as the layer gets deeper and with consequently lower time-consuming.

### Classification of cancer cells growth factor through DM-feature selection (Case study #5)

**Fig. 8:**
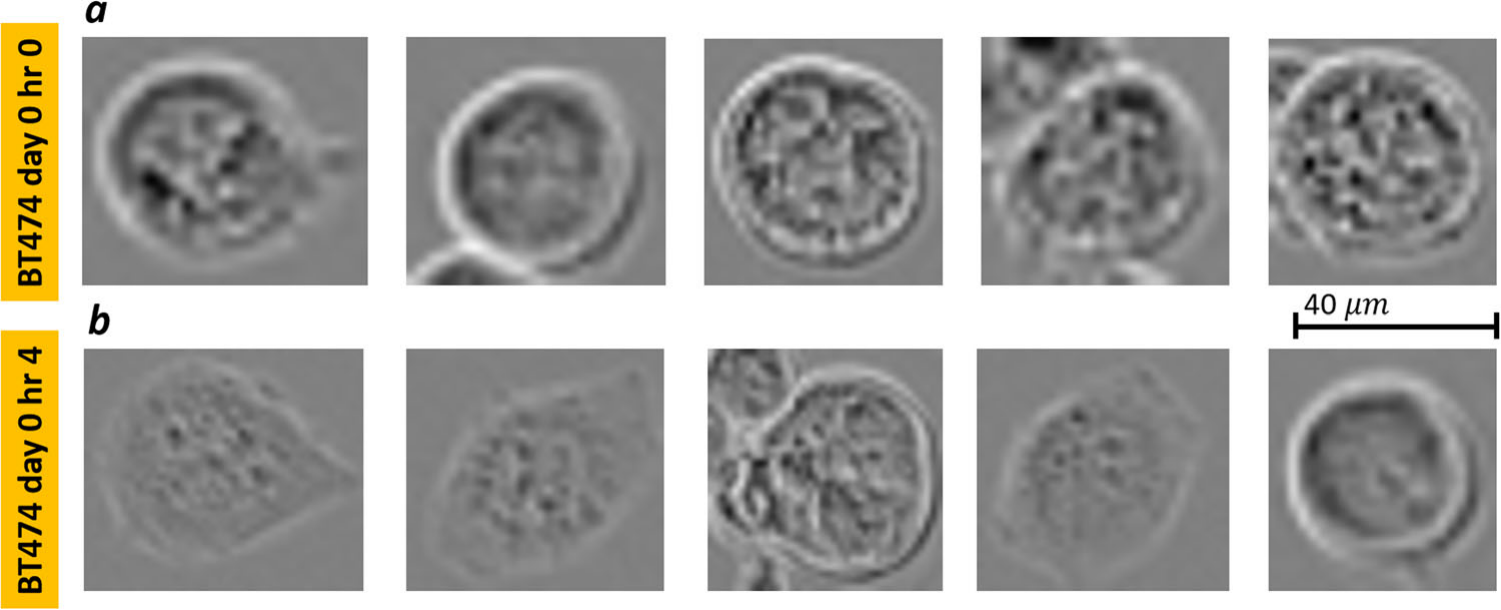
BT-474 cell images. Some examples of crops of BT-474 cells acquired at **a** day 0, hour 0 (top row), **b** BT-474 cells acquired at day 0, hour 4. Scale bar corresponding to 40 mm.

In this task, we reinforced the potential of using the DM-tool by implementing a classification task based on Support Vector Machine (SVM) trained over the deep features selected by the DM-tool. With this in mind, we selected a case study from the recently published LIVEcell labelled dataset^13^. In particular, with the aim to present a practical application in line with the examples used in this work, we selected BT-474 cells, breast cancer cells grown in rafts. The task was to recognize the cancer growth factor after 4 h. We then compared cells at day 0 with cells visualized after 4 h and applied the DM platform to select most discriminant and robust deep features for the task. Figure 8 shows five examples of cells from each group.

**Fig. 9:**
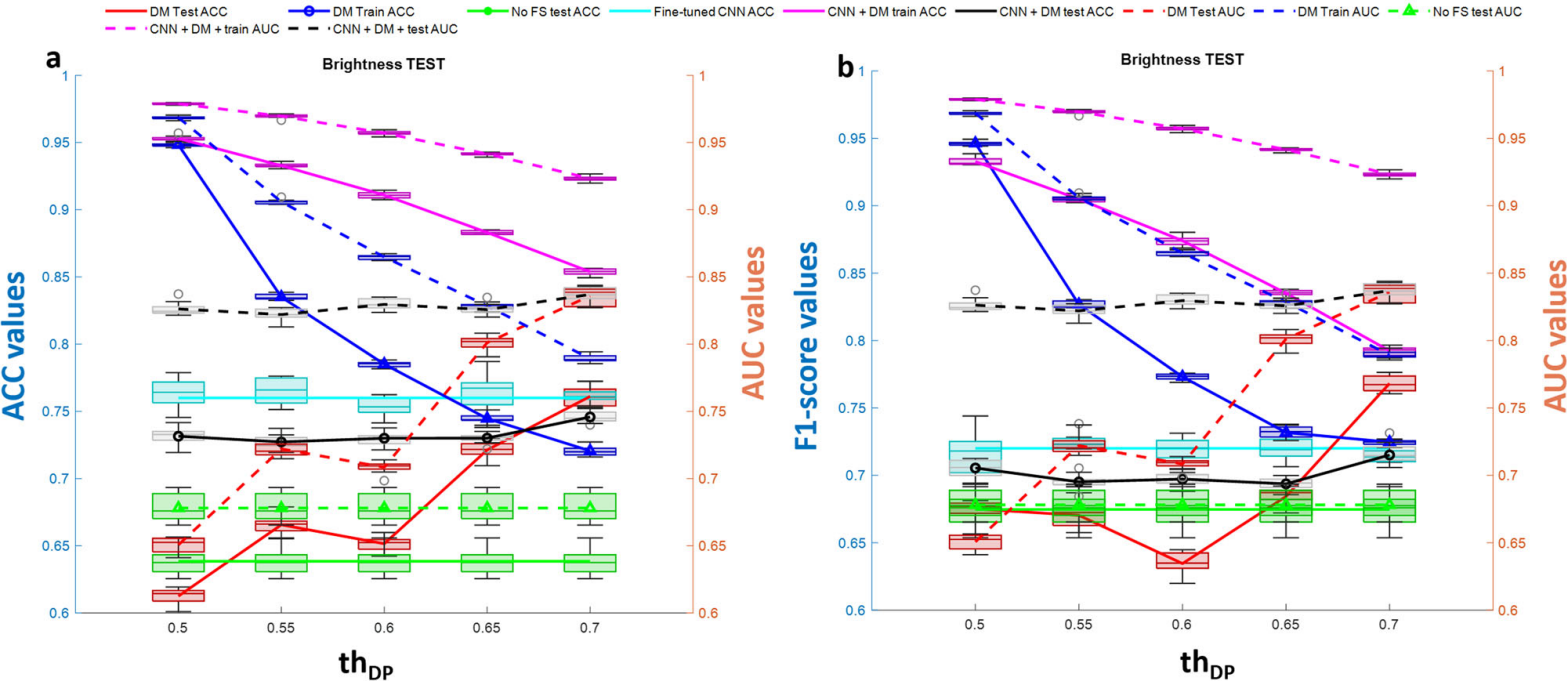
DM performance with brightness test. Classification performance of DM selection in combination with SVM classification model in terms of ACC (left *y*-axis panel **a**) and F1-score (left *y*-axis panel **b**) vs AUC (right *y*-axis) in recognizing BT-474 cells morphological changes across 4 h of culture at day 0. The independent test set of images has been modified according to the brightness test for the 2D scenario. Blue lines denote performance results calculated over the training set, red lines denote performance results calculated over the modified test set, green lines represent the performance results achieved by not selecting features, cyan line identifies the performance of the fine-tuned DENSENET201 network, black lines represent the performance of applying DM feature selection procedure to the features extracted by the fine-tuned DENSENET201 network. *n* = 10 repetitions were used to extract boxplot.

**Fig. 10:**
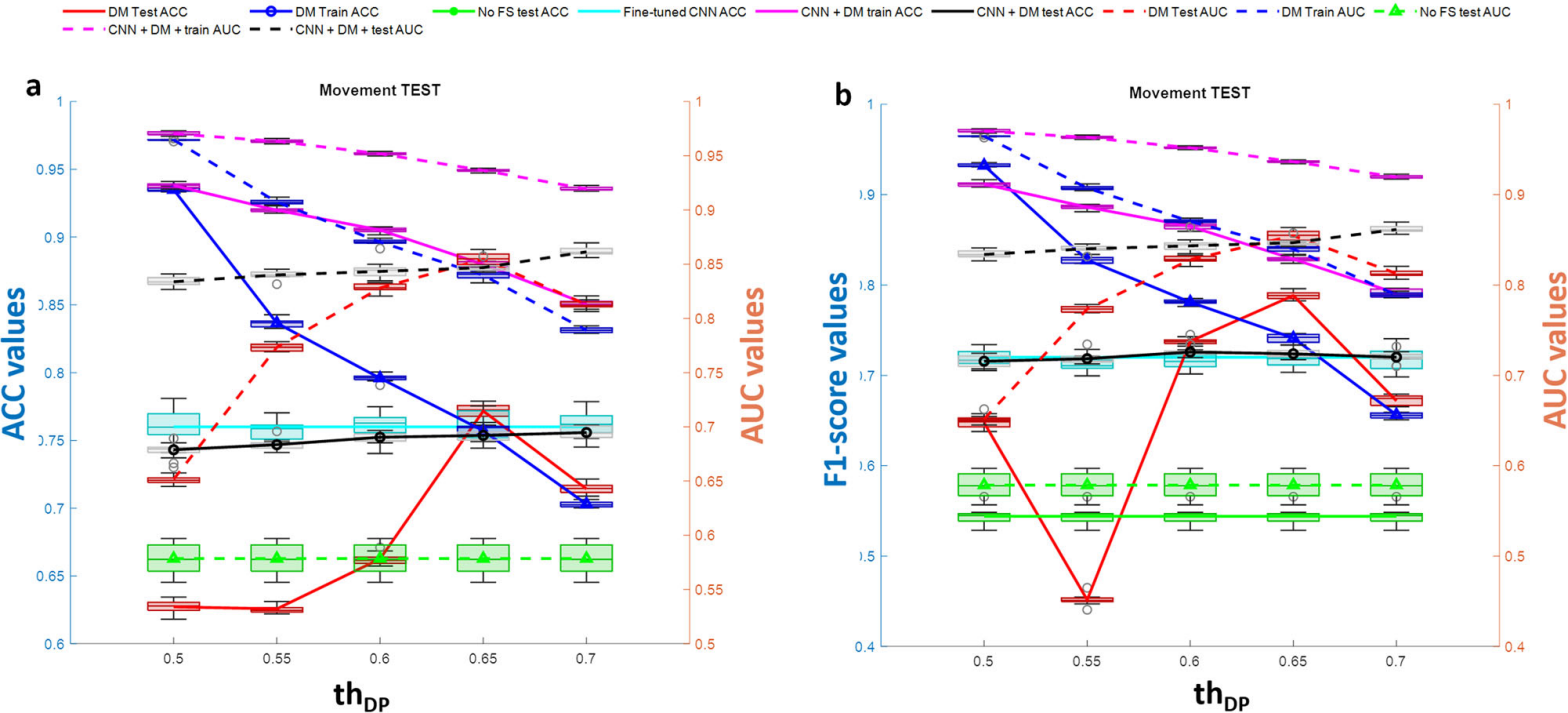
DM performance with movement test. Classification performance of DM selection in combination with SVM classification model in terms of ACC (left *y*-axis panel **a**) and F1-score (left *y*-axis panel **b**) vs AUC (right *y*-axis) in recognizing BT-474 cells morphological changes across 4 h of culture at day 0. The independent test set of images has been modified according to the movement test for the 2D scenario. Blue lines denote performance results calculated over the training set, red lines denote performance results calculated over the modified test set, green lines represent the performance results achieved by not selecting features, cyan line identifies the performance of the fine-tuned DENSENET201 network, black lines represent the performance of applying DM feature selection procedure to the features extracted by the fine-tuned DENSENET201 network. *n* = 10 repetitions were used to extract boxplot.

**Fig. 11:**
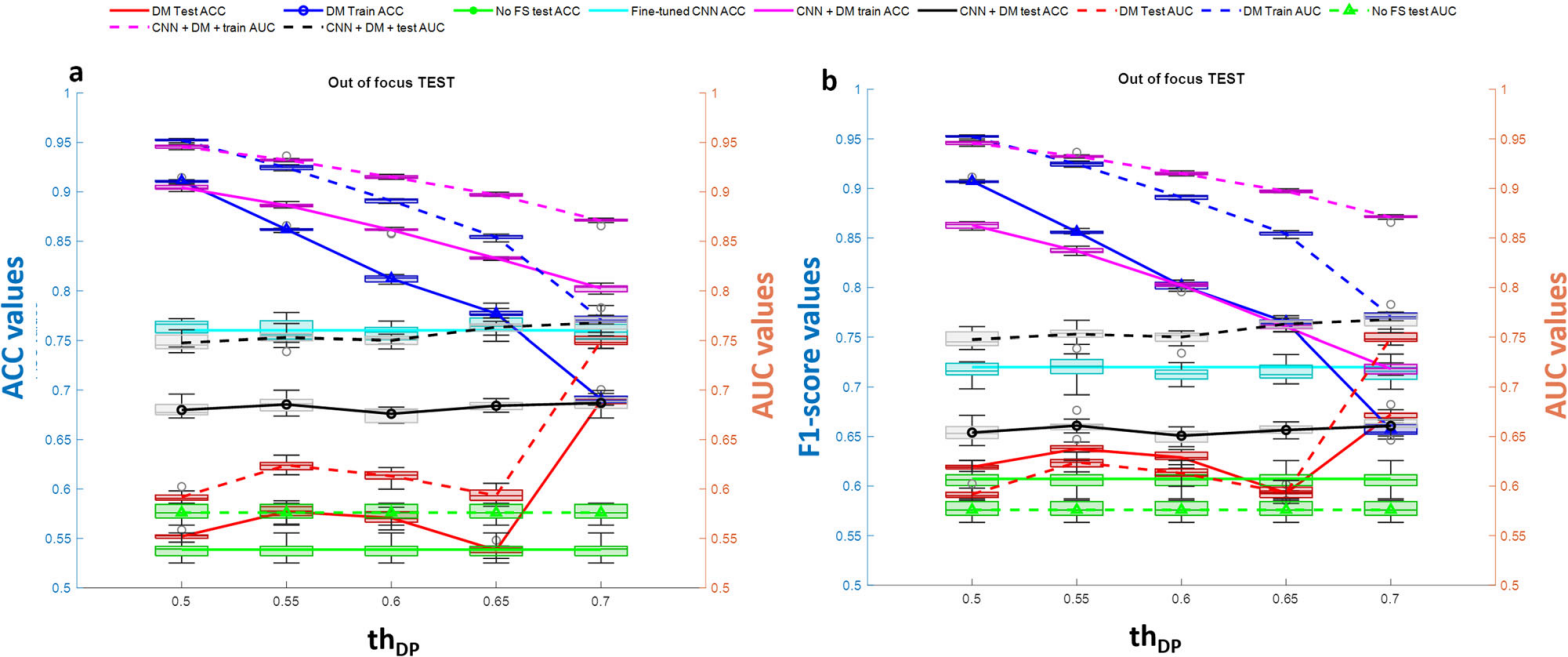
DM performance with out-of-focus test. Classification performance of DM selection in combination with SVM classification model in terms of ACC (left *y*-axis panel **a**) and F1-score (left *y*-axis panel **b**) vs AUC (right *y*-axis) in recognizing BT-474 cells morphological changes across 4 h of culture at day 0. The independent test set of images has been modified according to the out-of-focus test for the 2D scenario. Blue lines denote performance results calculated over the training set, red lines denote performance results calculated over the modified test set, green lines represent the performance results achieved by not selecting features, cyan line identifies the performance of the fine-tuned DENSENET201 network, black lines represent the performance of applying DM feature selection procedure to the features extracted by the fine-tuned DENSENET201 network. *n* = 10 repetitions were used to extract boxplot.

The annotations allow us to locate each cell and extract a Region of Interest (ROI) around it. According to the simulation results shown in Fig. 7, we selected the CNN Densenet201 with average pooling layer ‘average_pool5′ that exhibited the highest average DP values for all three tests. In order to create a challenging scenario, we performed the classification task of discriminating cells at day 0, hour 0 from cells at day 0, hour 4. To make the task realistic, avoiding overtraining of the model, we applied the perturbation tests 1–3 (luminance, movement, and out-of-focus) to the images in the test set, as provided by the LIVEcell dataset. We extracted all the 5554 ROIs annotated in the training dataset and test the 1912 ROIs from the test set after applying the random perturbations described in the method. The features extracted through the DM toolbox were then fed to an SVM with a linear kernel^20^ classification model with the aim to recognize cells morphology changes at the 4th hour of culture. Being the number of features selected very low (from 2 to 5 over 1920 extracted by the net), we also performed simulations by lowering the threshold value th_DP_ used for the selection of features according to their DP values over the training set. We considered the interval of values for th_DP_ equal to [0.5 ÷ 0.7] at a step of 0.05. Figures 9–11 (panel a) show the accuracy of classification (ACC) and the Area Under Curve (AUC) values and Figs. 9–11 (panel b) show the F1-score values related to the three variational tests for 2D transmission light environment. The independent test set of images has been modified according to Fig. 9a, b the brightness test for the 2D scenario, Fig. 10a, b the movement test for the 2D scenario, Fig. 11a, b the out-of-focus test for the 2D scenario. Blue lines denote performance results calculated over the training set, red lines denote performance results calculated over the modified test set, and green lines represent the performance results achieved by not selecting features. As it can be clearly noted, the opportunity to select the features, not only allows higher classification performance in terms of ACC, F1-score, and AUC but also strongly reduces the time required by the model to be trained. In both the three tests, training accuracy of classification, F1-score values, and AUC values decrease with a smaller number of features selected (higher *th*_DP_). At the same time, testing accuracy of classification, F1-score values, and AUC values increase. This phenomenon demonstrates a decreasing overtraining trend and an increasing system robustness to image variations. The low results achieved by not selecting the features (green lines) furthermore proved the importance of the use of the DM tool. As a final comparison, we fine-tuned the pre-trained DENSENET201 network over the same training set of images after the application of perturbations *test*1-*test*3 for *IM-ACQ-1* images. Training options for Stochastic Gradient Descent with Momentum (SGDM) optimization method were 10 *epochs* (1000 iterations per epoch), data *shuffle* at every epoch, mini-batch *size* equal to 10, 0.001 *initial learning rate*. Training took about 33 h on Matlab 2022b, using the GPU NVIDIA GeFORCE RTX and Intel Core i7, 9th generation. Also, we randomly selected 5000 training data from 16662 for computational time constraints. We then applied the tuned network to the test set used in the previous experiments. Results achieved in terms of ACC are shown in Figs. 9–11 as the cyan line. In addition, we also applied the DM feature selection criterion to the deep features extracted by the fine-tuned DENSENT201 network. Results are represented by the black lines. As it can be noted, the fine-tuned network is not sensible to the feature selection procedure, probably due to the tuning step. On the other hand, the generalization capability of the fine-tuned network remains quite low. Although results are comparable in terms of ACC and F1 scores, a training time of 33 h and the need to retrain the network in the presence of new perturbation tests, as needed, make DM tool the outperforming approach considering also that its reduced set of features with respect fine-tuned network is utilized as input for the classification models.

## Conclusions

The greatest goal for all those who develop data analysis techniques for medical applications is to see their work used in a real context. Unfortunately, the transition from the excellent results obtained in the laboratory, for example, in a hospital, is anything but easy. In the real measurement scenario, many variables are not controlled, and their variations can lead to not acceptable performances. Such an issue is even more critical when it is addressed in the context of deep learning features due to the lack of a priori known relationship between the black-box descriptors (deep features) and the phenotypic properties of the biological entities under study. In this work, we have introduced a software platform called Deep-Manager, that counteracts this limitation by analyzing the performance and sensibility of each feature to different disturbances. The potentiality of the proposed approach has been validated in five different case studies and different simulated artifacts, evidencing superior performances with respect to the standard solutions.

## Methods

Deep-Manager platform allows users to perform specific sensitivity tests to their own images dataset to select the most appropriate features for the specific classification task. Sensitivity tests aim to detect which features extracted from ad hoc algorithms (handcrafted) or from a pre-specified Deep Learning network through transfer learning approach are more sensitive to external quantities and phenomena that are acquisition-specific. Among the existing vast panorama of acquisition devices and experimental set-up, with the aim to prove the effectiveness of the proposed method, we selected three of the most used practical contexts in the field of biological image analysis: 2D transmission light time-lapse microscopy, 3D phase-contrast time-lapse microscopy, and 3D fluorescence time-lapse microscopy. The implemented sensitivity tests are therefore thought for those contexts. However, the list of possible tests of the Deep-Manager platform could be enlarged in the future to other fields such as histopathological imaging or indirect immunofluorescence. For this reason, in the remainder, we will indicate the present release as Deep-Manager 1.0 version. Link: https://github.com/BEEuniroma2/Deep-Manager. All the data required to reproduce the figures are in the Supplementary Data 1 file.

### Deep-Manager 1.0 considered imaging modality

Regarding the imaging modality the actual version of DM-tool considered three distinct imaging modalities (Supplementary Notes 1.1): IM-ACQ-1 *(2D TL time-lapse microscopy*, *Supplementary Notes**section)*, IM-ACQ-2 (*3D phase-contrast TL time-lapse microscopy)*, IM-ACQ-3 (*3D fluorescence time-lapse microscopy*). Each modality identifies specific tests related to the equipment and experimental conditions used.

### Deep-Manager 1.0 available artifacts

For the modality IM-ACQ-1 we implemented “Brightness artifact”, “Stage multi-positioning artifact”, “Out-of-focus artifact”. Case studies #2 and #5 refer to this scenario. For the modality IM-ACQ_2 we included tests “Brightness variation”, “Local-out of focus”, and “Gel texture variation”. Case study #3 refers to such scenario. Finally, for the modality IM-ACQ-3, we included “culture medium autofluorescence”, “photobleaching”, and “fluorescence saturation”. Case studies #1 and #4 refer to such modality. Additional mathematical details can be found in the Supplementary Notes (Sections 1.1, *IM-ACQ-1 – IM-ACQ-2*, Supplementary Figs. 1–9).

### Features available

The DM platform allows two distinct modalities: 1. handcrafted intensity and texture features 2. Deep-features from Deep Transfer Learning (DTL) algorithm. Users with programming skills may also add customized functions with specific additional features. A geometric descriptors extractor would require a preliminary segmentation step in order to extract the shape of each cell. By default, the platform proposes some well-known intensity and texture descriptors that are computed over the original image (or the image subjected to perturbations). The list of available intensity descriptors is: average intensity, median intensity, the standard deviation of the intensity, minimum intensity, 10th percentile of the intensity, 25th percentile of the intensity, 75th percentile of the intensity, 90th percentile of the intensity, maximum intensity, entropy of the intensity^21^. Regarding the texture descriptors, DM platform includes Haralick features^22^ and Histogram of Oriented Gradient features (HoG)^23^. Further details can be found in the references and in Section 1.2 of Supplementary Notes “*Handcrafted features”*. By selecting different deep layers, the input image is encoded into a different number of descriptors from detailed representation (higher layers) to coarser encoding (very deep layers). By default, the DM platform includes several well-known deep learning architectures: ResNET101^16^, VGG19^17^, NasNETLarge^18^, and DenseNET201^19^. Each network presents so-called pooling layers, that reduce the dimensions of data by combining the outputs of neuron clusters at one layer into a single neuron in the next layer^16,24^.

### Software design and utilization

The Deep-Manager platform has been realized in Python 3.8 open-source language in Anaconda framework. The overall platform architecture has been thought for different levels of expertise. A text file is fed the DM software including a list of parameters and related range values to be used in the artifacts implementation and application. A unique text file is available for all the tests so that the user may repeatedly run the platform by modifying a unique SETTING file. Advanced users may also modify the tests or add a new one by properly including the setting parameters in the SETTING file. The main steps of the DM functionalities are: (1) the user is first asked to select the practical scenario to work (such selection allows the platform to save final selection results into a specific file numbered according to the test number (e.g., 2D TL microscopy, 3D Phase Contrast TL microscopy, or 3D Fluorescence). All the tests available for the selected modality are applied; (2) the user is then asked to select the SETTING text file to load the DM configuration. The parameters used are listed in the Supplementary Notes, Section *Algorithm Parameter*. The file also includes the name of the network used for the transfer learning and the layer used to extract the features, if applicable. Specific details are provided in the Methods for each test. (3) the user is then asked to select the path where the training dataset of images is stored. Details can be found in the DM Guide https://github.com/BEEuniroma2/Deep-Manager; (4) the user is asked to select the handcrafted or the DTL modality. As a consequence, if the handcrafted selection is chosen, the platform automatically calculates a set of texture and intensity features. If DTL is selected, the platform reads the setting information regarding which network and layer to choose in the SETTING file mentioned above. DM applies the perturbations according to the tests described above and computes the features before and after the perturbation; (5) The user may visualize perturbation effects on images selected at random. It is also possible to visualize in a 2D plot values of DP vs SENSITIVITY for the selected and for unselected features; finally, (6) the user is then asked to select a directory containing two validation datasets on which to select the features for a discrimination task. All the image formats are allowed,.jpeg,.tiff,.png etc. Selected features are then computed for the validation dataset and saved in separate repository variable to be used in a classification task. The user may also save the modified set of training images for further usage. The selection process works as follows; using the two values achieved for each descriptor *f*_*i*_, i.e., *f*_*i0*_ and *f*_*imod*_, before and after the perturbations, the software derives the individual Discriminant Power (DP) values as follows:

1 $${{{{{\rm{DP}}}}}}_{i0}=\,{{{{{\rm{max }}}}}}\left(1-{{{{{\rm{AUC}}}}}}\,{(\,{f}_{i0})}_{{{{{{\rm{class}}}}}}1}^{{{{{{\rm{class}}}}}}2},{{{{{\rm{AUC}}}}}}\,{(\,{f}_{i0})}_{{{{{{\rm{class}}}}}}1}^{{{{{{\rm{class}}}}}}2}\right)$$

2 $${{{{{\rm{DP}}}}}}_{imod}=\,{{{{{\rm{max }}}}}}\left(1-{{{{{\rm{AUC}}}}}}\,{(\,{f}_{imod})}_{{{{{{\rm{class}}}}}}1}^{{{{{{\rm{class}}}}}}2},{{{{{\rm{AUC}}}}}}\,{(\,{f}_{imod})}_{{{{{{\rm{class}}}}}}1}^{{{{{{\rm{class}}}}}}2}\right)$$

and then, it computes the Sensitivity (SENS) of descriptor *f*_*i*_ to the added perturbation as follows:

3 $${{{{{\rm{SENS}}}}}}\,(\,{f}_{i})=\left|\frac{{{{{{\rm{DP}}}}}}_{imod}-{{{{{\rm{DP}}}}}}_{i0}}{{{{{{\rm{DP}}}}}}_{i0}}\right|$$

where ${{{{{\rm{AUC}}}}}}{({f}_{i0})}_{{{{{\rm{class1}}}}}}^{{{{{\rm{class2}}}}}}$ indicates the Area Under the Receiving Operating Characteristic (ROC) curve^25^ of feature *f*_*i*0_ in discriminating *class1* from *class2*. We consider here DP equal to the maximum value between 1-AUC and AUC, being AUC a way to quantify the discrimination capability of a descriptor in a binary classification problem^17^. With respect to the AUC value, the DP of each feature has been chosen due to its invariance to the “feature-to-label” direct or inverse relationship. In other words, either high or low AUC values (both indicative of highly discriminant capability) correspond to high DP values.

**Fig. 12:**
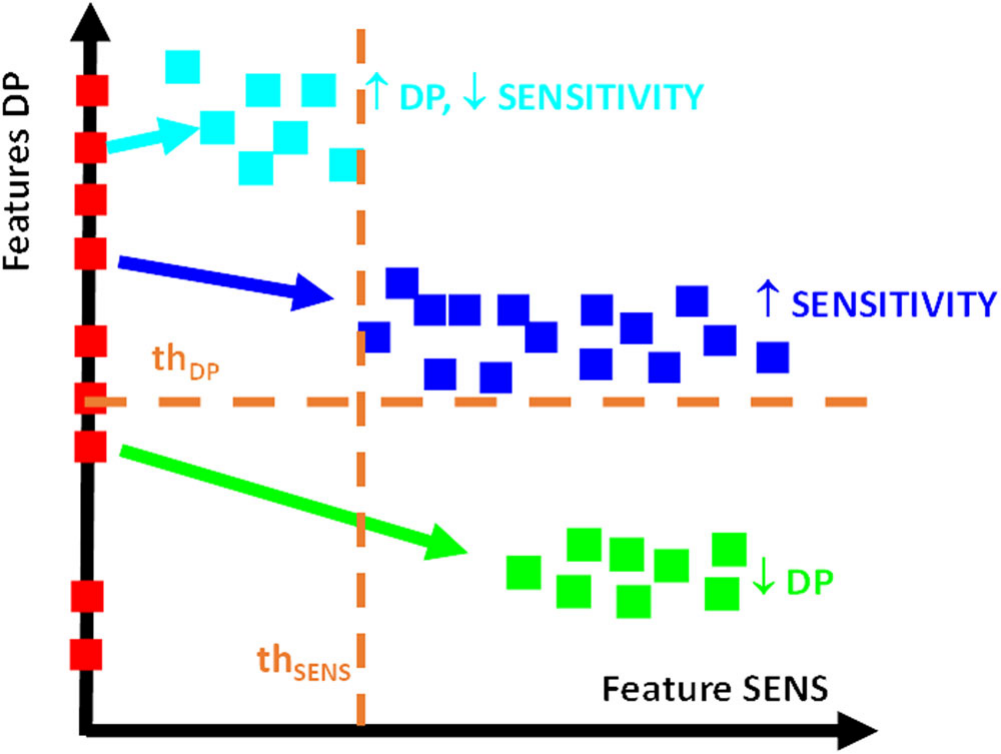
A visual example of the Deep-Manager possible outcome. Red markers locate the DP values for descriptors extracted using transfer learning before image modification. Sensitivity values are assumed to be zero being calculated before the application of the test. Cyan markers locate the (SENS, DP) values for each descriptor with high DP and low SENS. Blue markers locate the (SENS, DP) values for each descriptor with high SENS. Green markers locate the (SENS, DP) values for descriptors with low DP.

The software applies a threshold value *th*_DP_ to classify descriptors according to the DP values and a threshold value *th*_SENS_ to classify descriptors according to the sensitivity values. In light of this, descriptors are classified into different regions: *high DP* and *low SENS* (those selected) having DP higher than *th*_DP_ and sensitivity lower than *th*_SENS_ (cyan markers in Fig. 12)*, high SENS*, i.e., those rejected due to the high sensitivity larger than *th*_SENS_ to the artifact (blue markers in Fig. 12), and *low DP* smaller than *th*_DP_, i.e., those rejected because of their low discriminant power (green markers in Fig. 12).

The threshold values are loaded in the SETTING text file and may be modified by the user since they strongly depend on the application. A sketch of the possible Deep-Manager outcome is shown in Fig. 10. Being DP values in the range [0.5,1], admissible values of *th*_*DP*_ are in the range [0.6–0.8] according to the discrimination capability of the features extracted in the specific case. Regarding sensitivity, it is standardly in the range of [0.05–0.1] being it the expected variation of the DP before and after perturbation. When the threshold *th*_DP_ is too high or *th*_SENS_ is too small, and no feature is selected, then the tool gives an alert to the user to choose different threshold values.

#### Case Study #1: Fluorescence microscopy videos of chemotherapy-induced death of MDA-MB 231 breast cancer cells, in presence of a green fluorescent apoptosis reporter

An inverted Leica DMi8 equipped with a Retiga R6 camera and Lumencor SOLA SE 365 light engine, using a 5X objective, has been used to acquire Time-lapse images. The filter cubes used were TXRed (excitation filter 560/40 nm, emission filter 630/75 nm, dichroic mirror 585 nm) and GFP (excitation filter 470/40 nm, emission filter 525/50 nm, dichroic mirror 495 nm). A live fluorescent dye (CellTrace, red) was used to selectively pre-stain the cancer cells before cultures on-chip. To monitor apoptotic death, a live fluorescent reporter for caspase activity (CellEvent Caspase-3/7, green) was added to the on-chip culture medium. The red channel was then used to locate cells^10^ while the transposition on the green channel of the cancer cell position allowed to monitor green emission signal and, therefore, death events. Breast cancer cells (BT474 cell line, representative of HER2 + breast cancer subtype) were co-cultured in 3D biomimetic collagen gels, within microfluidic devices, with immune cells (PBMCs, peripheral blood mononuclear cells from healthy donors), with or without the addition of targeted immunotherapy, the trastuzumab (brand name Herceptin). With the aim to demonstrate the advantage of using DM tool in common practice, we extracted handcrafted features related to green emission and compared the case of standard practice with the use of DM tool.

#### Case study #2: TL microscopy videos of PC3 human prostate cancer cells, moving in a 2D environment in presence of the chemotherapeutic drug etoposide

In the case of 2D TL time-lapse microscopy experiments, good feature selection is of pivotal importance to exclude those features varying according to unpredictable changes such as luminance drift, flickering, focus changes over time, etc., that may hopelessly hamper data interpretation. With the aim to test the validity of the proposed Deep-Manager platform in such a context, we analyzed cancer cells before and after exposure to the chemotherapeutic drug etoposide, a topoisomerase II inhibitor blocking cell DNA replication that deeply affects cell motility, shape, and granularity over time, characteristics that may be easily misinterpreted if subjected to luminance drift, flickering, etc. Briefly, PC-3 human metastatic prostate cancer cells (ATCC) were grown in RPMI 1640 medium, supplemented with 10% fetal bovine serum, 1% L-glutamine (2 mM), and 1% penicillin/streptomycin (100 IU/mL) (Euroclone), at 37 °C in a humidified atmosphere of 5% CO2 in the air. In each experiment, 40,000 cells/mL were seeded in 35-mm Petri dishes (Euroclone). Seventy-two hours post-seeding, cells were treated with the chemotherapeutic drug etoposide (Sigma-Aldrich), a topoisomerase II inhibitor blocking cell DNA replication, at the final concentrations of 0 and 5 μM and immediately analyzed for time-lapse. Images were acquired via a custom small-scale inverted microscope at one frame per minute, with 6 h of total experimental time. In the presented results, we considered the two extreme conditions, 0 and 5 μM.

#### Case Study #3: Phase-contrast TL microscopy videos of immune cells moving in a 3D collagen gel inside microfluidic tumor-on-chip devices that mimic the tumor microenvironment

Breast cancer cells (BT474 cell line, representative of HER2 + breast cancer subtype) were co-cultured in 3D biomimetic collagen gels, within microfluidic devices, with immune cells (PBMCs, peripheral blood mononuclear cells from healthy donors), without or with the addition of targeted immunotherapy, the trastuzumab (brand name Herceptin). For details, see the original biological publication^12^. In this use case, we demonstrated how deep features’ discrimination capability of the effect of targeted immunotherapy in breast tumors is influenced by image alterations and compared results with those achieved using standard features selection approaches using diverse deep learning architectures.

#### Case Study #4: Fluorescence microscopy videos of cancer cells forced to undergo apoptosis by cytotoxic T cells in the 3D tumor-on-chip

Lung cancer cells (IGR-Pub) were co-cultured in 3D biomimetic collagen gels, within microfluidic devices, without or with immune cells (autologous cytotoxic T cells, clone P62). CellEvent Caspase-3/7 Green Detection Reagent (Thermofisher, #C10423) was added to the medium in order to visualize the cells undergoing apoptosis in the green channel. For details, see the original biological publication^10^. In this use case, we demonstrated how deep features’ discrimination capability of the T-cell cytotoxic effect in lung tumors is influenced by image alterations and compared results with those achieved using standard features selection approaches and diverse deep learning architectures.

#### Case Study #5. Phase contrast TL microscopy static images of BT-474 breast cancer cells from the recently presented public dataset LIVECell

With the aim to demonstrate the potential of selecting deep features through the proposed DM tool, we selected a case study from the recently published LIVECell labeled dataset^13^. In particular, with the aim to present a practical application in line with the examples used in this work, we selected BT-474 cells, breast cancer cells grown in rafts. The cell lines were purchased from ATCC and were cultured as per suppliers’ recommendations. Several wells for each cell type were seeded in 96-well plates (Corning) and imaged over the course of 5 days, every 4 h using an Incucyte S3 Live-Cell Analysis system (Sartorius) equipped with its standard CMOS camera (Basler acA1920-155 um). Such equipment avoided the presence of the phase annulus found in conventional Zernike phase images. Phase-contrast TL images were acquired using a ×10 objective from two positions in each well and then cropped into four equally sized images (704 × 520 pixels corresponding to 0.875 × 0.645 mm2). The images were then annotated by a team of experts.

### Statistics and reproducibility

Statistical significance was evaluated by implementing a Student t-test. Repeatability was assured by 10-randomly subsampling using hold-out cross validation approach (e.g., Figs. 9–11). The number of samples used for each statistical analysis was enclosed in the corresponding figure caption.

### Reporting summary

Further information on research design is available in the Nature Portfolio Reporting Summary linked to this article.

## Supplementary information

Reporting Summary

Supplementary Information

Supplementary Data 1

Description of Additional Supplementary Data

## Data Availability

The authors declare that data supporting the findings of this study are available within the paper [and its supplementary information files]. Any other support and request can be submitted at the corresponding author email: martinelli@ing.uniroma2.it or filling the form that can be found at https://web.bee.uniroma2.it/our-contacts/. Images belonging to the LIVECell dataset can be downloaded at https://sartorius-research.github.io/LIVECell/. See also ref. ^13^.

## Code availability

Deep-Manager software and example images to run the code can be freely downloaded at https://github.com/BEEuniroma2/Deep-Manager.
