# Supplementary material for: Deep-Manager: a versatile tool for optimal feature selection in live-cell imaging analysis
Source: Commun Biol. 2023 Mar 3;6:241. doi: 10.1038/s42003-023-04585-9 (PMC9984362; doi:10.1038/s42003-023-04585-9)
Supplement: Supplementary file 2 — Supplementary Information [file 42003_2023_4585_MOESM2_ESM.pdf]

Supplementary Information

Deep-Manager: a versatile tool for optimal feature selection in live-cell imaging analysis

A. Mencattini[1,2*], M. D'Orazio[1,2*], P. Casti[1,2], M. C. Comes[1,2], D. Di Giuseppe[1,2], G. Antonelli[1,2], J. Filippi[1,2], F. Corsi[2,3], L. Ghibelli[3], I. Veith[4,5], C. Di Natale[1], M.C. Parrini[4,5], E. Martinelli[1,2,#]

[1]Department of Electronic Engineering, University of Rome Tor Vergata, 00133, Rome, Italy
[2]Interdisciplinary Center for Advanced Studies on Lab-on-Chip and Organ-on-Chip Applications (IC-LOC), University of Rome Tor Vergata, 00133 Rome, Italy.
[3] Department of Biology, University of Rome Tor Vergata, 00133, Rome, Italy
[4]Institut Curie, Centre de Recherche, Paris Sciences et Lettres Research University, 75005 Paris, France
[5] Inserm U830, Stress and Cancer Lab, 75005 Paris, France
#Corresponding author: Eugenio Martinelli, martinelli@ing.uniroma2.it
*These authors contribute equally

Summary

This Supplemental Material describes in detail the software architecture of the Deep-Manager platform. The effectiveness of the Deep-Manager application is proved using five case studies herein specified. In addition, steps and tests conducted on the platform are explained. We also presented a glossary of terms for those unfamiliar with Deep Learning, microscopy, and microfluidic devices terminology and a list of algorithm parameters used in the tool. Experimental results obtained by the proposed platform and comparison with state-of-the-art methodologies for feature selection are also presented and discussed.

Table of contents

Glossary **3**

Algorithm Parameters **4**

Supplementary Note 1. Deep-Manager overall architecture **5**

Supplementary Note 1.1 Deep-Manager 1.0 available artifacts *5*

IM-ACQ-1 5

IM-ACQ-2 7

IM-ACQ-3 9

Supplementary Note 1.2 Feature available *11*

Handcrafted features 11

Deep-features 11

Supplementary Note 1.3 Software design and utilization *12*

Supplementary References **14**

Glossary

Transmission light time-lapse microscopy: optical microscopy illumination techniques in which sample illumination is transmitted (i.e., illuminated from below and observed from above) white light, and contrast in the sample is caused by attenuation of the transmitted light in dense areas of the sample.

Phase-contrast transmission light time-lapse microscopy: optical microscopy technique that converts phase shifts in light passing through a transparent specimen to brightness changes in the image.

Fluorescence microscopy: optical microscope that uses fluorescence instead of, or in addition to, scattering, reflection, and attenuation or absorption, to study the properties of organic or inorganic substances.

Time-lapse microscopy: time-lapse photography applied to microscopy in which microscope image sequences are recorded and then viewed at a greater speed to give an accelerated view of the microscopic process.

Microfluidics: a set of technologies used for the manipulation of small fluid volumes (μL, nL, pL) within artificially fabricated microsystems, and cell culture, which involves the maintenance and growth of cells in a controlled laboratory environment.

Deep Learning: a part of a broader family of machine learning methods based on artificial neural networks with representation learning, in which the term *deep* refers to the use of multiple layers in the network.

Transfer Learning: a research problem in machine learning that focuses on storing knowledge gained while solving one problem and applying it to a different but related problem.

Handcrafted features: features referring to properties derived by applying various unsupervised algorithms to extrapolate the information present in the image, distinct from features extracted from a neural network architecture. In a broader sense, the term also refers to features extracted "by hand" and not "by a machine".

Region-of-Interest: a region in which it is supposed to be an object, i.e., a cell, of interest.

Feature Discriminant Power: a numerical value that measures the capability of a single feature to discriminate objects belonging to two different categories.

Feature Sensitivity: a numerical value that measures the difference in the feature discriminant power when computed on two distinct variations of the same image.

Feature Selection: the process of selecting a subset of relevant features for use in classification model construction.

Feature Ranking: the process of ranking all the available features for further feature selection.

Algorithm Parameters

$\boldsymbol{l}_0$ *luminance value for brightness variation in 2D transmission light imaging*

$\boldsymbol{l}_{\min}$ *minimum value of the range for* $\boldsymbol{l}_0$

$\boldsymbol{l}_{\max}$ *maximum value of the range for* $\boldsymbol{l}_0$

$\boldsymbol{th_S}$ *threshold value for saturation in fluorescence saturation*

$\boldsymbol{l_x^{min}}$ *minimum value* for the *x-shift term in stage multipositioning artifact in 2D TL microscopy imaging*

$\boldsymbol{l_x^{max}}$ *maximum value* for the *x-shift term in stage multipositioning artifact in 2D TL microscopy imaging*

$\boldsymbol{l_y^{min}}$ *minimum value* for the *y-shift term in stage multipositioning artifact in 2D TL microscopy imaging*

$\boldsymbol{l_y^{max}}$ *maximum value* for the *y-shift term in stage multipositioning artifact in 2D TL microscopy imaging*

$\boldsymbol{\alpha_{min}}$ *minimum angle value for the rotation in stage multipositioning artifact in 2D* TL microscopy *imaging*

$\boldsymbol{\alpha_{max}}$ *maximum angle value for the rotation in stage multipositioning artifact in 2D* TL microscopy *imaging*

$\boldsymbol{R}_{\min}$ *minimum radius of the disk-shaped spatial filter for out-of-focus artifact*

$\boldsymbol{R}_{\max}$ *maximum radius of the disk-shaped spatial filter for out-of-focus artifact*

$\boldsymbol{\lambda_{min}}$ *minimum attenuation of the gel-texture pattern artifact to impose in 3D phase-contrast TL imaging*

$\boldsymbol{\lambda_{max}}$ *maximum attenuation of the gel-texture pattern artifact to impose in 3D phase-contrast TL imaging*

$\boldsymbol{\theta_{min}}$ *minimum angle of rotation of the gel-texture pattern artifact to impose in 3D phase-contrast TL imaging*

$\boldsymbol{\theta_{max}}$ *maximum angle of rotation of the gel-texture pattern artifact to impose in 3D phase-contrast TL imaging*

$\boldsymbol{b}_r$ *ratio between the fluorescence emitted in the bleached object and in the original image in 3D fluorescence imaging*

$\boldsymbol{th_S}$ *threshold luminance value for saturation artifact in 3D fluorescence imaging*

$\boldsymbol{th_{DP}}$ *threshold value for feature selection according to the DP values after artifact addition*

$\boldsymbol{th_{SENS}}$ *threshold value for feature selection according to the SENS values after artifact addition*

Supplementary Note 1. Deep-Manager overall architecture

The Deep-Manager platform allows users to perform specific sensitivity tests on their own images dataset to select the most appropriate features for the specific classification task. Sensitivity tests aim to detect which features extracted from ad hoc algorithms (handcrafted) or a pre-specified Deep Learning network through a transfer learning approach are more sensitive to external quantities and phenomena that are acquisition-specific. Among the vast panorama of acquisition devices and experimental set-up, to prove the effectiveness of the proposed method, we selected three of the most used practical contexts in the field of biological image analysis: 2D transmission light time-lapse microscopy, 3D phase-contrast time-lapse microscopy, and 3D fluorescence time-lapse microscopy. The implemented sensitivity tests are therefore thought for those contexts. However, the list of possible tests of the Deep-Manager platform could be enlarged in the future to other fields such as histopathological imaging or indirect immunofluorescence. For this reason, in the remainder, we will indicate the present release as Deep-Manager 1.0 version. Link : https://github.com/BEEuniroma2/Deep-Manager

Supplementary Note 1.1 Deep-Manager 1.0 available artifacts

IM-ACQ-1

Test 1) Brightness artifact

The brightness test randomly applies an overall brightness change to each image in the dataset by numerical adding a luminance value to all the pixels in the image within a user-defined range. In this way, we simulate the drift in the luminance level of the lamp or the sudden changes in acquisition set-up, for example, due to external light sources' influence (opening of the incubator, changes in room settings) [1].

By indicating with $I(x, y)$ each single image entering the brightness artifact experiment and by denoting with l_0 a generic luminance level in the range [$-l_{min}$, l_{max}], then the modified image $I_b(x,y)$ is obtained by

$$I_b(x, y) = \max(0, \min(I(x, y) + l_0, 1)) \quad (1)$$

where the minimum and the maximum operators are needed to assure the final range of the image being in [0,1] as it is expected.

An example of the brightness artifact is shown in Fig. S1.

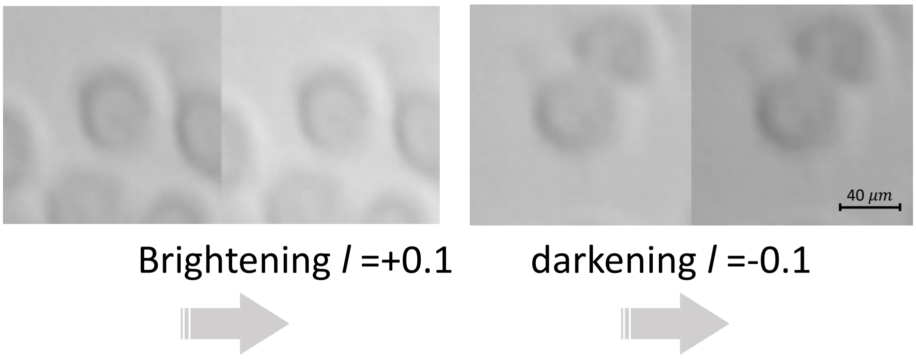

Supplementary Figure 1*. Visual example of brightening (left) with luminance value l=0.1 and darkening (right) with luminance value l=-0.1. Scale bar is equal to 40 μm.*

Test 2) Stage multi-positioning artifact

Stage multi-positioning is a frequent practice in time-lapse microscopy experiments for saving time and conducting parallel experiments. However, the exact repositioning of the stage is not always easy to achieve, and a very small object movement can be observed (in the order of 1 pixel, i.e., less than 1 μm). To simulate such an effect, the Cartesian domain of the image is randomly rotated and translated of an angle, and a shift term on the x-axis and y-axis in a given range defined by the user.

By indicating with x and y the original coordinate system, with l_x and l_y the shift terms in x and y respectively, $l_x \in [-l_x^{min}, l_x^{max}]$, $l_y \in [-l_y^{min}, l_y^{max}]$, and with α the rotation angle, $\alpha \in [\alpha_{min}, \alpha_{max}]$, then we define the modified coordinate system as

$$\begin{cases} x_\alpha = x \cdot \cos(\alpha) + y \cdot \sin(\alpha) + l_x \\ y_\alpha = x \cdot \sin(\alpha) - y \cdot \cos(\alpha) + l_y \end{cases} \quad (2)$$

where the centre of rotation is considered as the geometrical centre of the image.

An example of the movement artifact is shown in Fig. S2.

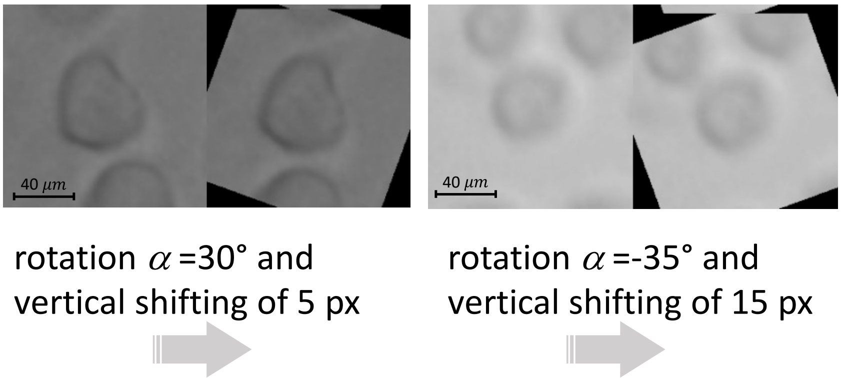

Supplementary Figure 2. *Visual example of rotation with α=30° ad vertical shift of 5 pixels (left) and rotation with α=-35° and vertical shift of 15 pixels (right). Scale bar is 40 μm.*

Test 3) Out-of-focus artifact

Experiments conducted over living samples are critical, especially for maintaining culture composition as stable as possible, i.e., oxygen and carbon dioxide concentration, temperature, and relative humidity. Evaporation of small quantities of liquid and/or related changes of compounds' concentration may create effects such as out-of-focus. Similarly, cell death or relevant changes in cell dimension may induce out-of-focus effects. Out-of-focus effects appear like a blurring phenomenon with decreasing cell details and increasing cell dimension. To simulate out-of-focus effects, we applied a disk-shaped spatial filter to each image, with a radius R in the range [R_{min}, R_{max}]. The Point Spread Function (PSF) of the disk-shaped filter is given by[2].

$$h(x, y) = \frac{1}{\pi R^2} \begin{cases} 1, & for \quad x^2 + y^2 \leq R^2 \\ 0, & otherwise \end{cases} \quad (3)$$

and the application of the filter over the image is achieved by convolution as follows:

$$I_o(x, y) = h(x, y) * I(x, y) \quad (4)$$

An example of the out-of-focus artifact is shown in Fig. S3.

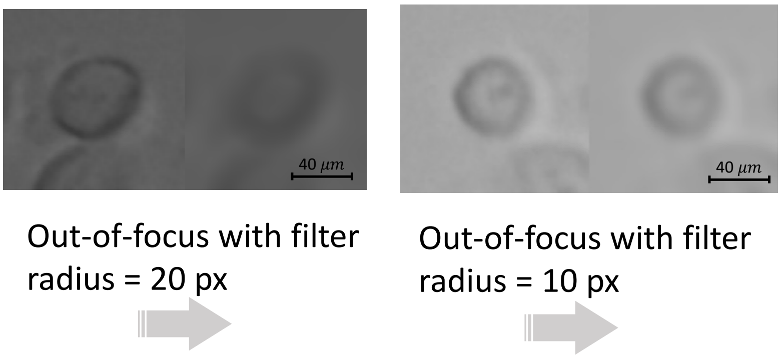

Supplementary Figure 3. *Visual example of out-of-focus with filtering radius = 20 pixels (left) and with radius=10 pixels (right). Scale bar is 40 μm.*

IM-ACQ-2

3D phase-contrast TL microscopy is an optical microscopy technique that converts phase shifts in light to brightness changes in the image. When light waves travel through a medium, their interaction causes the wave amplitude and phase to change in a manner dependent on the properties of the medium. Phase-contrast microscopy is particularly important in biology. It reveals many cellular structures that are invisible under a bright-field microscope. The phase-contrast microscope allows biologists to study living cells and how they proliferate through cell division without staining the cells, which requires additional preparation. And often the death of the cells.

Test 1) Brightness variation

Even if the phase-contrast imaging is much more robust to general brightness variation, it may occur especially as inter-experiment variations, namely across different experiments, using different microscopy devices. General brightness variation may also be an operator-dependent artifact due to the manual adjustment of the image contrast and luminance that may occur during the acquisition.

Brightness variation in phase-contrast imaging can be achieved through Eq. 1, by properly setting $l_{\min}$=0 and $l_{\max}$ to a quite low value since, on average, the background is very dark and the image can be only brightened. An example of the brightness artifact in phase-contrast imaging is shown in Fig. S4.

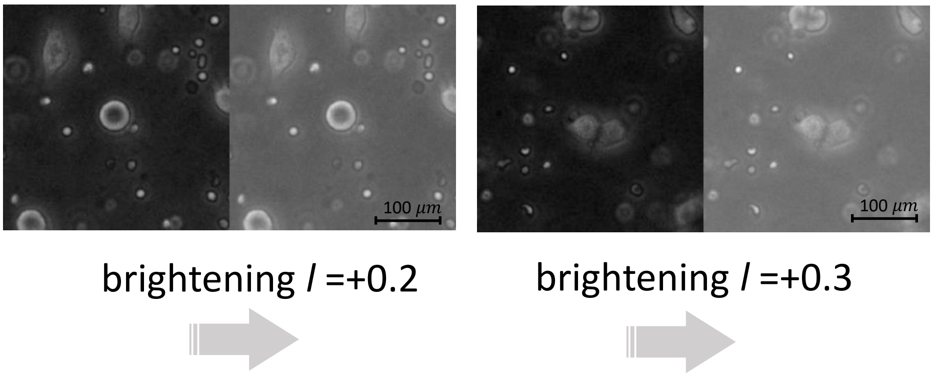

Supplementary Figure 4. *Visual example of brightness variation with level l = 0.2 (left) and l = 0.3 (right) in phase-contrast images. Scale bar is 100μm.*

Test 2) Local-out of focus
Phase-contrast imaging is usually used to acquire images from a 3D environment in which cells move in the space in every direction. To simulate such kind of local out-of-focus, we apply the filter in Eq. 3 with radius R in the range [10 ÷30] because, in the 3D domain, cells are free to move in a larger space.

An example of the local out-of-focus artifact in phase-contrast imaging is shown in Fig. S5.

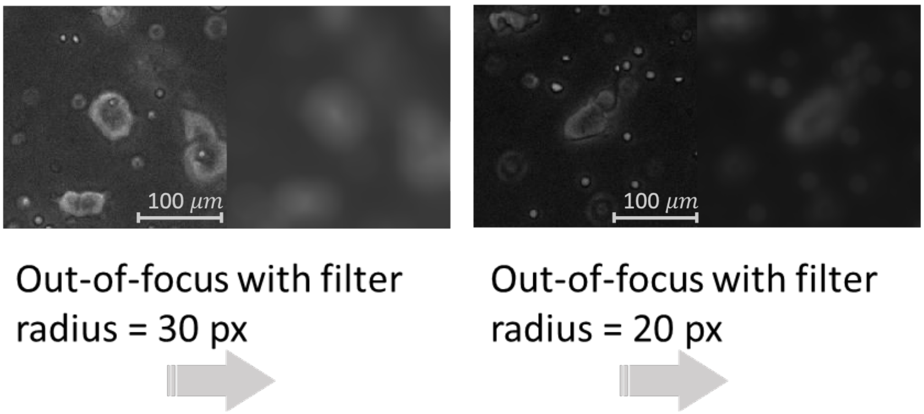

Supplementary Figure 5. *Visual example of out-of-focus with filtering radius = 30 pixels (left) and with radius=20 pixels (right). Scale bar is 100μm.*

Test 3) Gel texture variation
In living cell experiments, cells are cultured into matrices mainly composed of collagen (e.g., type I)[3], which is the major component of the tumor microenvironment and participates in cancer fibrosis. Collagen biosynthesis can be regulated by cancer cells through mutated genes, transcription factors, signaling pathways, and receptors; furthermore, collagen can influence tumor cell behavior through integrins, discoidin domain receptors, tyrosine kinase receptors, and some signalling pathways. By transmission electron microscope, collagen is appeared to be structured in oriented microfibers. When visualized in phase-contrast imaging, collagen fibers are almost invisible due to the practical diffraction limits close to 0.2 μm, but due to some heterogeneity, the collagen texture may be visible in some regions of the field of view. With the aim to simulate such an artifact, we generate a striped pattern that is then attenuated by a factor $\lambda \in [\lambda_{min}, \lambda_{max}]$ and over-imposed on each image. Orientation θ of the patterns is set randomly in the range [θ_{min}, θ_{max}]. An example of an artificial gel pattern image and related modifications is shown in Fig. S6 A).

An example of the image of the striped patterns and of the collagen pattern artifact in phase-contrast imaging is shown in Fig. S6 B.

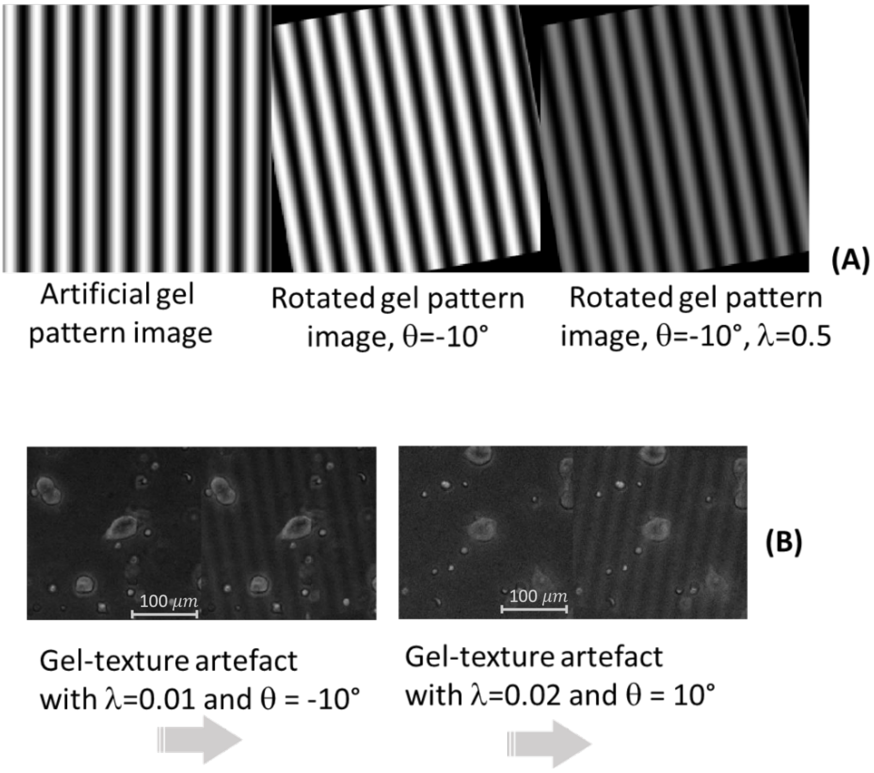

Supplementary Figure 6. *A) An example of the gel-pattern map, vertical, rotated of an angle θ = -10°, and attenuated with λ=0.5. B) Visual example of gel texture artifact applied with λ = 0.01 and θ = -10° (left) and λ = 0.02 and θ = 10° (right). Scale bar is 100μm.*

IM-ACQ-3

The fluorescence microscopy technique has become an essential tool in biology and the biomedical sciences due to the ability to extract information at a deeper scale with the respect to the traditional optical microscopy[4]. The application of an array of fluorochromes has made it possible to identify cells and sub-microscopic cellular components with a high degree of specificity[5]. In fact, the fluorescence microscope is capable of revealing the presence of a single molecule. Such phenomena are usually correlated with biological events such as cell death[4] or cell replication. Different artifact effects may occur in fluorescence microscopy, such as autofluorescence, photobleaching, and fluorescence saturation.

Test 1) Autofluorescence

Autofluorescence is the natural emission of light by biological structures such as mitochondria, lysosomes, elastin, and collagen[6] when they have absorbed light, and makes it complicated to distinguish the light originating from artificially added fluorescent markers[7]. Autofluorescence spectra are generally broad, extending over several hundred nanometers. Hence, its interference is often significant at the same emission wavelengths as GFP leading to low signal-to-noise ratios and loss of contrast and clarity in fluorescence microscope images.

Autofluorescence is observed in regions with no strong marker emission and may occur when applying local contrast enhancement approaches. Of course, there can be found non-cellular (i.e., background autofluorescence) as well as cellular autofluorescence. Due to the large variety and specificity of cellular autofluorescence sources[6], the DM tool includes the implementation of a background autofluorescence test. Further versions will consider the case of anisotropic subcellular autofluorescence phenomena.

Therefore, to simulate the presence of autofluorescence, we apply a brightness increase only to the object background in the channel devoted to the cell localization (usually the red). To do this, we first locate the cells stained in red, then extract the local background and apply a brightness increase to this latter one. Brightness increase is achieved by applying the method described in iv) only to the red channel that is used in the presented case study to stain lung tumor cells.

An example of the autofluorescence artifact image is shown in Fig. S7.

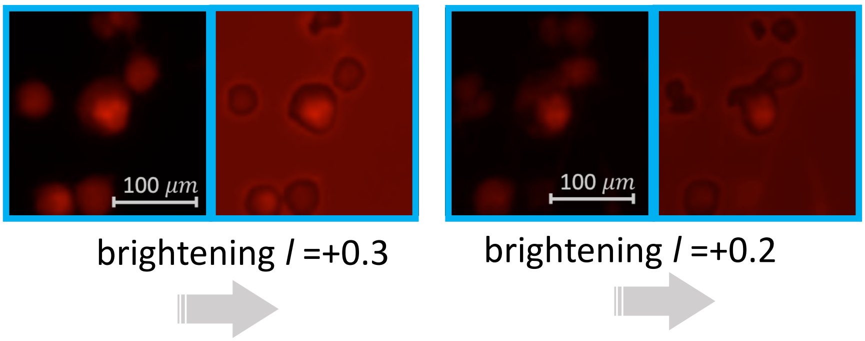

Supplementary Figure 7. *Visual example of the autofluorescence artifact shown in the red channel with* $l = 0.3$ *(left) and* $l = 0.2$ *(right). Scale bar is 100μm.*

Test 2) Photobleaching

Photobleaching (also *fading*) is the photochemical alteration of a fluorophore molecule leading it to a permanent disabling to fluoresce caused by non-specific reactions between the fluorophore and surrounding molecules[8]. In microscopy, photobleaching may complicate the observation of fluorescent molecules since they will eventually be destroyed by the light exposure necessary to stimulate them into fluorescing. Photobleaching also occurs when cells replicate after dividing the cytoplasm hence diluting the die. Even if photobleaching is also a method to reduce autofluorescence[6] due to the diverse decaying of GFP signal and background autofluorescence, it may also strongly affect the final achievements of the experiment. To simulate photobleaching, we decrease the red response (but the same holds for the green channel or both) of stained lung cancer cells taken from fluorescence time-lapse microscopy images. The background is assumed to remain dark (no autofluorescence mechanism is simultaneously present). Parameter variations are introduced by setting the percentage of emitted signal decreasing with respect to the value at the first frame. We define a so-called *bleaching ratio,* b_r, as the ratio between the average red response in the bleached image and in the image at the first frame. Future expansions of such test will consider, when time-lapse microscopy is available, exponential decaying bleaching phenomenon. An example of the photobleaching artifact image is shown in Fig. S8

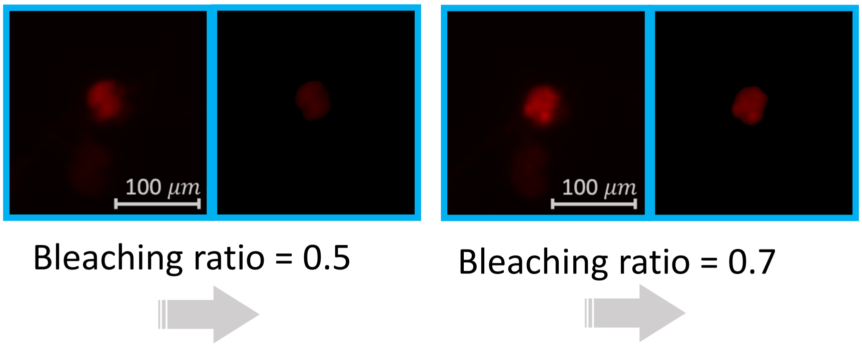

Supplementary Figure 8. *Visual example of the photobleaching artifact shown in the red channel with* $b_r = 0.5$ *(left) and* $b_r = 0.7$ *(right). Scale bar is 100μm.*

Test 3) Fluorescence saturation
Fluorescence saturation effects may impact the spatial resolution of time-lapse microscopy images and induce a loss of details in examining cell parts and functionalities. This can be observed, for example, in cancer cells stained for cell death[4]. To simulate such an artifact, we induce luminance saturation in green-stained cancer cells a certain time before they go into final death. In this way, the evolution of the cell death phenomenon is also biased towards a stronger effect.

Mathematically, denoting with I(x,y) the image in the red channel, then we apply the following piecewise linear operation to obtain the saturated image I_S(x,y)[2].

$$I_S(x,y) = \begin{cases} I(x,y), & I < th_S \\ 1, & I \geq th_S \end{cases} \tag{5}$$

where th_S is the threshold value considered for saturation effect. An example of the fluorescence saturation artifact image is shown in Fig. S9.

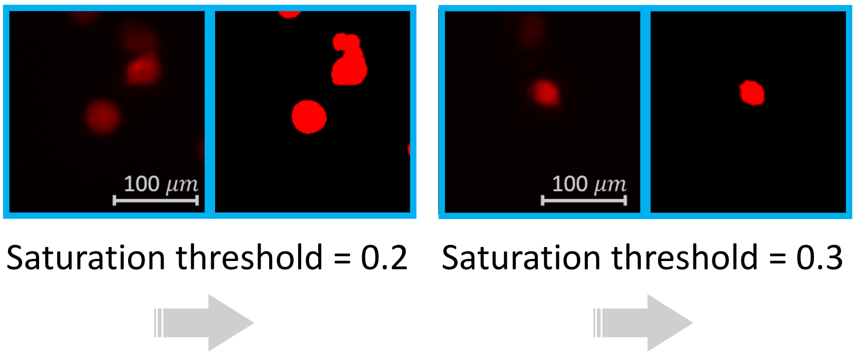

Supplementary Figure 9. *Visual example of the fluorescence saturation artifact shown in the red channel with* $th_s = 0.2$ *(left) and* $th_s = 0.3$ *(right). Scale bar is 100μm.*

Supplementary Note 1.2 Feature available

The DM platform allows two distinct modalities: 1. handcrafted intensity and texture features 2. Deep-features from Deep Transfer Learning (DTL) algorithm. Users with programming skills may also add customized functions with specific additional features.

Handcrafted features
By default, the platform proposes some well-known intensity and texture descriptors that are computed over the original image (or the image subjected to perturbations). The list of available intensity descriptors is: average intensity, median intensity, the standard deviation of the intensity, minimum intensity, 10th percentile of the intensity, 25th percentile of the intensity, 75th percentile of the intensity, 90th percentile of the intensity, maximum intensity, entropy of the intensity[2]. Regarding the texture descriptors, the DM platform includes Haralick features[9] and Histogram of Oriented Gradient features (HoG)[10]. Haralick features represent the statistics of the so-called gray-level co-occurrence matrix, a different rearrangement of the image information taking into account spatial dependence and local pixel similarity. The HoG features are a representation of the distribution of intensity gradients or edge directions. The image is divided into small connected regions, and for the pixels within each region, a histogram of gradient directions is compiled. The descriptors are the concatenation of these histograms.

Deep-features
Deep Transfer Learning (DTL) is an approach in deep learning (and machine learning) where knowledge is transferred from one model to another. We can use transfer learning to solve a particular task using an entire or part of a model already pre-trained on a different task. DTL can treat the pre-trained neural

network as a feature extractor by discarding the last fully-connected output layer. This approach allows for using a lightweight linear classification or regression model (Support Vector Machine, Linear Discriminant Analysis, Support Vector Regression, Multivariate Linear Regression[11] starting from the extracted features and allows the use of a network already trained for days or weeks on state-of-the-art machines. By selecting different deep layers, the input image is encoded into a different number of descriptors, from detailed representation (higher layers) to coarser encoding (very deep layers). By default, the DM platform includes several well-known deep learning architectures: ResNET101[12], VGG19[13], NasNETLarge[13], and DenseNET201[14]. Each network presents so-called *pooling layers* that reduce the data dimensions by combining the outputs of neuron clusters at one layer into a single neuron in the next layer[15]. The result of using a pooling layer and creating downsampled or pooled feature maps is a summarized version of the features detected in the input. They are useful thanks to the fact that small changes in the location of the feature in the input detected by the convolutional layer will result in a pooled feature map with the feature in the same location. This capability added by pooling is called the model's invariance to local translation.

Supplementary Note 1.3 Software design and utilization

The Deep-Manager platform has been realized in Python 3.8 open-source language in the Anaconda framework. The overall platform architecture has been thought for different levels of expertise. A text file is fed to the DM software, including a list of parameters and related range values to be used in the artifacts implementation and application. A unique text file is available for all the tests so that the user may repeatedly run the platform by modifying a unique SETTING file. Advanced users may also modify the tests or add a new one by properly including the setting parameters in the SETTING file.

In the following, we list the main steps of the DM functionalities.

STEP1. The user is first asked to select the practical scenario to work on (such selection allows the platform to save final selection results into a specific file numbered according to the test number (e.g., 2D TL microscopy, 3D Phase Contrast TL microscopy, or 3D Fluorescence). All the tests available for the selected modality are applied.

STEP2. The user is then asked to select the SETTING text file to load the DM configuration. The parameters used are listed in the *Algorithm Parameter* Section. The file also includes the name of the network used for the transfer learning and the layer used to extract the features, if applicable. Specific details are provided in the previous sections for each test.

STEP3. The user is then asked to select the path where the training dataset of images is stored. Details can be found in the DM Guide https://github.com/BEEuniroma2/Deep-Manager.

STEP4. The user is asked to select the handcrafted or the DTL modality. As a consequence, if the handcrafted selection is chosen, the platform automatically calculates a set of texture and intensity features. If DTL is selected, the platform reads the setting information in the SETTING file mentioned above. DM applies the perturbations according to the tests described above and computes the features before and after the perturbation.

STEP5. The user may visualize perturbation effects on images selected at random. It is also possible to visualize in a 2D plot values of DP vs. SENSITIVITY for the selected and unselected features. Using the two values achieved for each descriptor f_i, i.e., f_{i0} and f_{imod}, before and after the perturbations, the software derives the individual Discriminant Power (DP) values as follows:

$$DP_{i0} = \max\left(1 - AUC(f_{i0})_{class1}^{class2}, AUC(f_{i0})_{class1}^{class2}\right) \quad (6)$$

$$DP_{i\,mod} = \max\left(1 - AUC(f_{imod})_{class1}^{class2}, AUC(f_{imod})_{class1}^{class2}\right) \quad (7)$$

and then, it computes the Sensitivity (SENS) of descriptor f_i to the added perturbation as follows:

$$SENS(f_i) = \left|\frac{DP_{imod} - DP_{i0}}{DP_{i0}}\right| \quad (8)$$

where $AUC(f_{i0})_{class1}^{class2}$ indicates the area under the roc curve[16] of feature f_{i0} in discriminating *class1* from *class2*. The software applies a threshold value th_{DP} to classify descriptors according to the DP values and a threshold value th_{SENS} to classify descriptors according to the sensitivity values. In light of this, descriptors are classified into different regions: *high DP* and *low SENS* (those selected) having DP higher than th_{DP} and sensitivity lower than th_{SENS} (cyan markers in Fig. S10) *, high SENS*, i.e., those rejected due to the high sensitivity larger than th_{SENS} to the artifact (blue markers in Fig. S10), and *low DP* smaller than th_{DP}, i.e., those rejected because of their low discriminant power (green markers in Fig. S10). The threshold values are loaded in the SETTING text file and may be modified by the user since they strongly depend on the application. The user should tune the two hyperparameters, th_{DP} *and* th_{SENS}, in order to select a non-empty and small set of features (usually from 10 to 100). Typical values for DP and SENS thresholds are th_{DP} in [0.6 - 0.7], and th_{SENS} in [0.1 - 0.2], respectively.

STEP6. The user is then asked to select a directory containing two validation datasets on which to select the features for a discrimination task. All the image formats are allowed, .jpeg, .tiff, .png etc. Selected features are then computed for the validation dataset and saved in a separate repository variable to be used in a classification task. The user may also save the modified set of training images for further usage.

Supplementary References

1. Sung, M. A Checklist for Successful Quantitative Live Cell Imaging in Systems Biology. *Cells* **2**, 284–293 (2013).

2. Gonzalez, R. C. & Woods, R. E. *Digital Image Processing*. (Addison-Wesley Longman Publishing Co., 2001).

3. Xu, S. *et al.* The role of collagen in cancer : from bench to bedside. *J. Transl. Med.* **17**, 1–22 (2019).

4. Veith, I. *et al.* Apoptosis mapping in space and time of 3D tumor ecosystems reveals transmissibility of cytotoxic cancer death. *PLoS Comput. Biol.* **17**, 1–23 (2021).

5. Lichtman, J. W. & Conchello, J. Fluorescence microscopy. *Nat. Methods* **2**, 910–919 (2005).

6. Knight, A. W. & Billinton, N. Distinguishing GFP from cellular autofluorescence. *Biophotonics Int.* **8**, 42–51 (2001).

7. Stockert, J. C. & Blázquez-Castro, A. *Fluorescence microscopy in life sciences*. (Bentham Science Publishers, 2017).

8. Greenbaum, L., Rothmann, C., Lavie, R. & Malik, Z. Green Fluorescent Protein Photobleaching : a Model for Protein Damage by Endogenous and Exogenous Singlet Oxygen. *Biol. Chem.* **381**, 1251–1258 (2000).

9. Haralick, R. M., Shanmugam, K. & Dinstein, I. Textural Features for Image Classification. *IEEE Trans. Syst. Man Cybern.* (1973) doi:10.1190/segam2015-5927230.1.

10. Dalal, N., Triggs, B. & Europe, D. Histograms of Oriented Gradients for Human Detection. in *2005 IEEE computer society conference on computer vision and pattern recognition (CVPR'05)* (2005).

11. Duda, R. O., Hart, P. E. & Stork, D. G. *Pattern Classification*. (John Wiley & sons, 2001).

12. He, K., Zhang, X., Ren, S. & Sun, J. Deep Residual Learning for Image Recognition. *Proc. IEEE Comput. Soc. Conf. Comput. Vis. Pattern Recognit.* (2015).

13. Bhandary, A. *et al.* Deep-learning framework to detect lung abnormality – A study with chest X-Ray and lung CT scan images ☆. *Pattern Recognit. Lett.* **129**, 271–278 (2020).

14. Huang, G., Zhuang, L., van der Maaten, L. & Weinberger, K. W. Densely Connected Convolutional Networks. in *CVPR 2017* (2017).

15. Brownlee, J. A gentle introduction to pooling layers for convolutional neural networks. https://machinelearningmastery.com/pooling-layers-for-convolutional-neural-networks/ (2019).

16. Hanley, J. A. & Mcneil, B. J. The Meaning and Use of the Area under a Receiver Characteristic (ROC) Curve. *Radiology* **143**, 29–36 (1982).
