# Supplementary material for: Deep-Manager: a versatile tool for optimal feature selection in live-cell imaging analysis
Source: Commun Biol. 2023 Mar 3;6:241. doi: 10.1038/s42003-023-04585-9 (PMC9984362; doi:10.1038/s42003-023-04585-9)
Supplement: Supplementary file 4 — Description of Additional Supplementary Data [file 42003_2023_4585_MOESM4_ESM.docx]

**Description of Additional Supplementary Files**

**File name:** Supplementary Data 1

- **Description:** A directory is created with this file, containing txt and xls files of the data to reproduce figures.
